# STK25 Loss Augments Anti‐PD‐1 Therapy Efficacy by Regulating PD‐L1 Stability in Colorectal Cancer

**DOI:** 10.1002/advs.202503891

**Published:** 2025-07-29

**Authors:** Xiaowen Qiao, Pu Xing, Hao Hao, Jiangbo Chen, Lin Song, Yifan Hou, Xinying Yang, Kai Weng, Jie Chen, Pin Gao, Tongkun Song, Hong Yang, Tianqi Liu, Yumeng Ran, Bo Chen, Wei Zhao, Jiabo Di, Zaozao Wang, Jun Zhang, Xiangqian Su, Beihai Jiang

**Affiliations:** ^1^ Key Laboratory of Carcinogenesis and Translational Research (Ministry of Education) Department of Gastrointestinal Surgery IV Peking University Cancer Hospital & Institute Beijing 100142 China; ^2^ Department of Radiation Oncology Radiation Oncology Key Laboratory of Sichuan Province Sichuan Clinical Research Center for Cancer Sichuan Cancer Hospital &Institute Sichuan Cancer Center Affiliated Cancer Hospital of University of Electronic Science and Technology of China Chengdu Sichuan 610041 China; ^3^ School of Basic Medical Sciences Peking University Health Science Center Beijing 100191 China; ^4^ Department of Gastrointestinal Surgery Peking University Cancer Hospital (Inner Mongolia Campus) & Affiliated Cancer Hospital of Inner Mongolia Medical University Hohhot 010010 China; ^5^ Department of Pathology Peking Union Medical College Hospital Peking Union Medical College and Chinese Academy of Medical Science Beijing 100730 China; ^6^ Ningxia Clinical Research Institute People's Hospital of Ningxia Hui Autonomous Region Ningxia Medical University Yinchuan 750002 China; ^7^ Department of Immunology School of Basic Medical Sciences NHC Key Laboratory of Medical Immunology Medicine Innovation Center for Fundamental Research on Major Immunology‐related Diseases Peking University Beijing 100191 China; ^8^ State Key Laboratory of Holistic Integrative Management of Gastrointestinal Cancers Department of Gastrointestinal Surgery IV Peking University Cancer Hospital & Institute Beijing 100142 China

**Keywords:** colorectal cancer, immunotherapy, PD‐L1, STK25, tumor immune evasion

## Abstract

Tumor immune evasion is intricately linked to malignant tumor progression and contributes to the failure of anti‐cancer immunotherapy. Serine/threonine protein kinase 25 (STK25) has been previously implicated in the progression of various neoplastic diseases. However, the function of STK25 in the colorectal cancer (CRC) microenvironment remains unclear. Here, it is demonstrated that STK25 global knockout (STK25^−/−^) mice and STK25‐knockout tumor‐bearing mice exhibited enhanced effectiveness of anti‐PD‐1 immunotherapy, which leads to significant tumor suppression with increased recruitment of CD8^+^ T cells. Mechanistically, STK25 deficiency increased PD‐L1 protein levels by regulating PD‐L1 K48‐linked ubiquitination in a NEDD4‐dependent manner. Moreover, CRC patients with low STK25 expression are more responsive to immune checkpoint blockade (ICB) therapy compared to those with high STK25 levels. Taken together, the findings reveal a critical role of STK25 for regulating PD‐L1 protein stability in tumor immune evasion, and suggest that targeting STK25 may provide a potential approach to increase sensitivity to the ICB treatment in patients with CRC.

## Graphical abstract

This study demonstrates that STK25 depletion facilitates CRC immune escape through the inhibition of PD‐L1 Ser283 phosphorylation‐mediated ubiquitination, and promotes tumor growth. Furthermore, these findings identify STK25 as a potential therapeutic target to trigger antitumor immunity and promote immunotherapy efficacy.

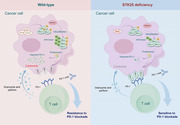

## 1 Introduction

Colorectal cancer (CRC) is one of the most prevalent malignancies in the digestive system,^[^ ^1^ ^]^ characterized by high rates of relapse, metastasis, and heterogeneity, making clinical management challenging.^[^ ^2^ ^]^ Immune checkpoint blockade (ICB), especially the anti‐PD‐1 therapy, has significantly changed the landscape of CRC therapy.^[^ ^3^, ^4^ ^]^ However, a large number of patients still fail to respond to the ICB monotherapy due to primary or acquired drug resistance.^[^ ^5^, ^6^, ^7^ ^]^ Hence, understanding the mechanisms of immune evasion in CRC and developing new combinatorial immunotherapy strategies alongside ICB are crucial for improving patient outcomes.As one of the primary mechanisms of tumor immune evasion, the PD‐1/PD‐L1 pathway prevents T cell activation, resulting in the immune escape of the tumor.^[^ ^8^ ^]^ It has been reported that manipulation of the PD‐1/PD‐L1 signaling in the tumor microenvironment or induction of PD‐L1 expression is related to immune escape and tumor progression.^[^ ^9^ ^]^ For instance, ADORA1 deficiency significantly contributes to the development of non‐small cell lung cancer (NSCLC) through the ATF3‐PD‐L1 axis, while ZNF652 inhibition can exacerbate triple‐negative breast cancer by upregulating the PD‐L1 expression.^[^ ^10^, ^11^ ^]^ PD‐L1 expression on tumor cells is widely recognized as a marker for assessing clinical response to ICB therapy.^[^ ^12^ ^]^ Furthermore, researchers have found that the development of inhibitors targeting upstream regulatory molecules of PD‐L1 can enhance the efficacy of ICB therapy.^[^ ^13^ ^]^ However, the mechanisms regulating PD‐L1 in CRC remain poorly understood. Therefore, it is vital to identify key molecules that can regulate PD‐L1 levels and potentially serve as targets for combination immunotherapy in CRC.

STK25, or Serine/Threonine Kinase 25, is a critical regulator of lipid metabolism, glucose homeostasis, and cell polarization.^[^ ^14^, ^15^, ^16^, ^17^, ^18^ ^]^ Increased expression of STK25 aggravated lipid storage, as well as impaired glucose tolerance and insulin sensitivity.^[^ ^14^, ^16^ ^]^ Loss of the STK25 disrupts neuronal migration during mouse embryogenesis.^[^ ^17^ ^]^ There is increasing evidence that STK25 is a pivotal molecule in various diseases. Inhibition of STK25 could significantly slow the progression of metabolic diseases, including diabetes and non‐alcoholic fatty liver disease.^[^ ^16^, ^18^ ^]^ Dysregulation of STK25 levels has been shown to be associated with neurological disorders, particularly cerebral cavernous malformations and Alzheimer's disease.^[^ ^19^, ^20^ ^]^ Recent studies have identified the role for STK25 in tumor progression. STK25 promotes cancer progression in hepatocellular carcinoma (HCC) by enhancing YAP1 activation.^[^ ^21^ ^]^ Our previous study demonstrated that STK25 inhibits CRC cell proliferation by regulating GOLPH3‐dependent mTOR signaling and STAT3‐mediated autophagy, respectively.^[^ ^22^, ^23^ ^]^ Notably, through bioinformatics analysis based on the TIMER database, Xu et al. found that STK25, as a ROS‐related gene, may be associated with immune cell infiltration in HCC.^[^ ^24^ ^]^ However, the role of STK25 in modulating the tumor immune microenvironment remains unclear.

In this study, we highlight the crucial role of STK25 in tumor immune regulation. We found that STK25 deficiency accelerated the progression of CRC in STK25^−/−^ mice by reducing CD8^+^ T cell infiltration. Further results indicated that STK25 depletion upregulated the expression of PD‐L1 via the phosphorylation‐ubiquitination pathway. Notably, loss of STK25 sensitized CRC to ICB therapy. Overall, our findings uncover the effect of STK25 in regulating PD‐L1 and propose a novel therapeutic strategy for CRC by targeting STK25 in combination with ICB therapy.

## 2 Results

### 2.1 STK25 Global Knockout Exacerbates the Development of AOM/DSS‐Induced CRC in Mice

**Figure 1:**
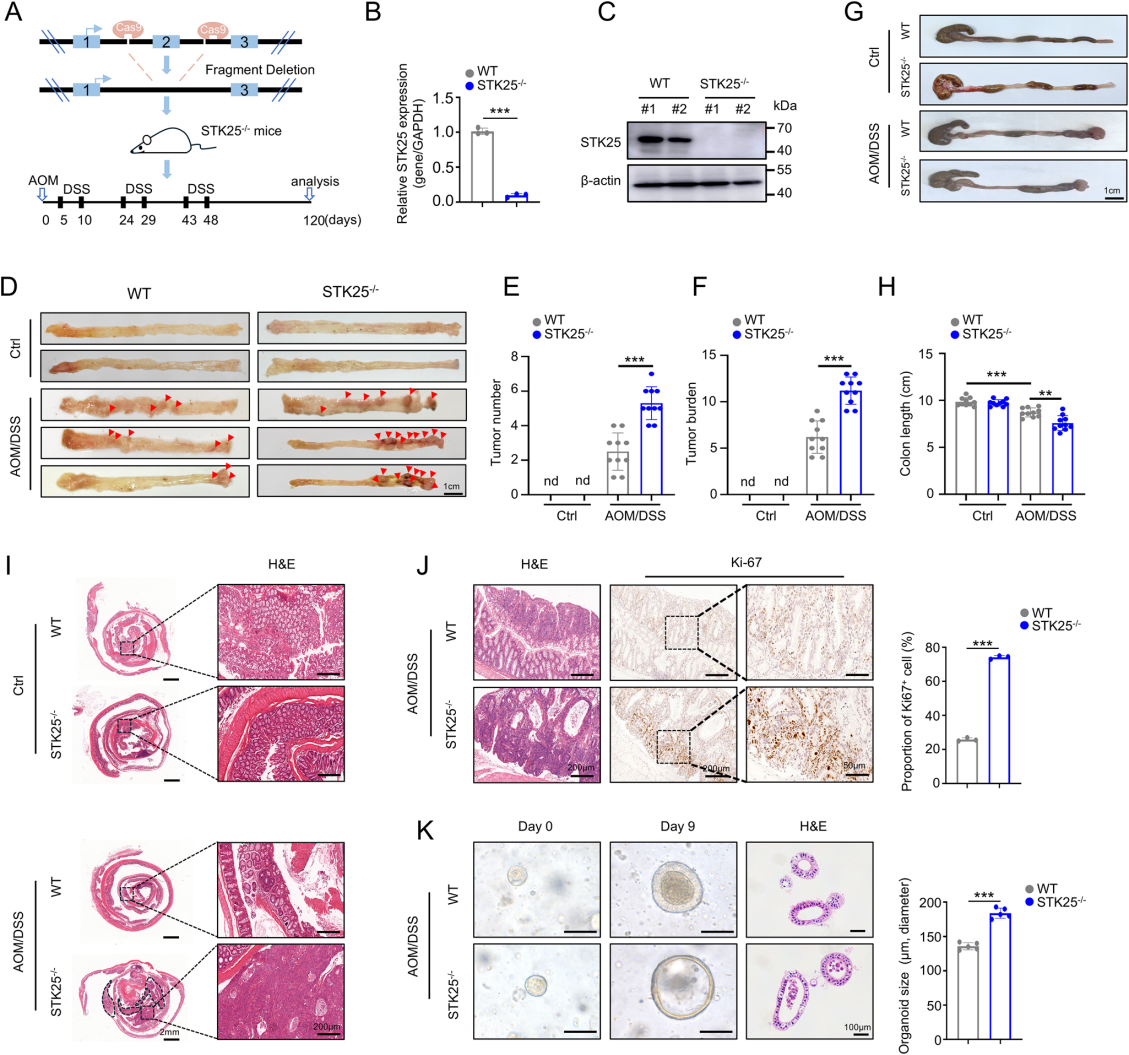
Global knockout of STK25 promotes AOM/DSS‐induced colorectal tumorigenesis in mice. A) Schematic diagram depicting the method for the generation of whole‐body STK25 knockout (STK25^−/−^) mice and the procedure for the AOM/DSS‐induced CRC model. B,C) qRT‐PCR (B) and Western blot (C) analysis of STK25 knockout efficiency in colon tissues from WT mice and STK25^−/−^ mice. D–F) Representative images of the opened colon from WT and STK25^−/−^ mice (n = 10 per group) treated with AOM/DSS or distilled water (D) and quantification of tumor number (E), as well as tumor burden (F). Scale bar, 1 cm. G,H) Representative macroscopic images of intestinal tissue (G) and quantification of intestinal length (H) are shown. Scale bar, 1 cm. I,J) H&E (I) and IHC staining of Ki‐67 (J, left panel) on colon sections from WT and STK25^−/−^mice (n = 3 per group) treated with AOM/DSS or distilled water. The percentage of Ki67‐positive cells was quantified (J, right panel). Scale bars, 2 mm, 200 µm, and 50 µm. K) Representative bright field images and H&E of primary CRC organoids from mice (n = 3 per group). Scale bar, 100 µm. The difference between two groups was determined by a Student's *t*‐test. ^**^ *p* < 0.01; ^***^ *p* < 0.001; nd, not detect.

To explore the function of STK25 in CRC, we constructed STK25 global knockout (STK25^−/−^) mice using CRISPR/Cas9 and induced CRC in both wild‐type and STK25^−/−^ mice by administering azoxymethane (AOM) and dextran sulfate sodium (DSS) (**Figure** **1**A). The genotype of the mice was confirmed by PCR amplification of genomic DNA (Figure S1A, Supporting Information), and the knockout efficiency of STK25 in intestinal tissues was verified by qRT‐PCR and Western blot analysis (Figure 1B,C). During AOM/DSS treatment, we monitored the alterations in body weight and fecal occult blood in mice. Although both groups exhibited body weight loss and increased fecal occult blood scores during the period of DSS treatment, no significant differences were observed between the groups (Figure S1B,C, Supporting Information). In addition, no remarkable histopathological differences were detected in several organ tissues including brain, lung, heart, liver, stomach, spleen, kidney, testis, uterus, skeletal muscle, and skeleton between wild‐type and STK25^−/−^ mice (Figure S1D, Supporting Information).

The mice were sacrificed at 120 days after CRC induction for tumor analysis. As expected, the AOM/DSS‐induced group developed intestinal tumors, while no tumors were observed in the intestinal tissue of the control group (Figure 1D), confirming successful induction of the mouse CRC models. Meanwhile, STK25^−/−^ mice exhibited a higher number of tumors and increased tumor burden compared to WT mice after AOM/DSS treatment (Figure 1D–F). Furthermore, compared with the WT mice, global depletion of STK25 in the AOM/DSS group significantly reduced the colon length of mice (Figure 1G,H), correlating with CRC severity. Consistent with these findings, histopathological analysis further revealed more tumor lesions and a higher percentage of Ki67‐positive cells in the STK25^−/−^ mice compared to WT mice as demonstrated by H&E and IHC staining (Figure 1I,J). This was further confirmed by constructing the intestinal organoids from the CRC mouse model (Figure 1K). Collectively, our findings indicated that loss of STK25 enhanced the formation of AOM/DSS‐induced CRC in vivo.

### 2.2 Loss of STK25 Remodels Tumor Microenvironment and Impairs CD8+T Cell Infiltration in Murine CRC

**Figure 2:**
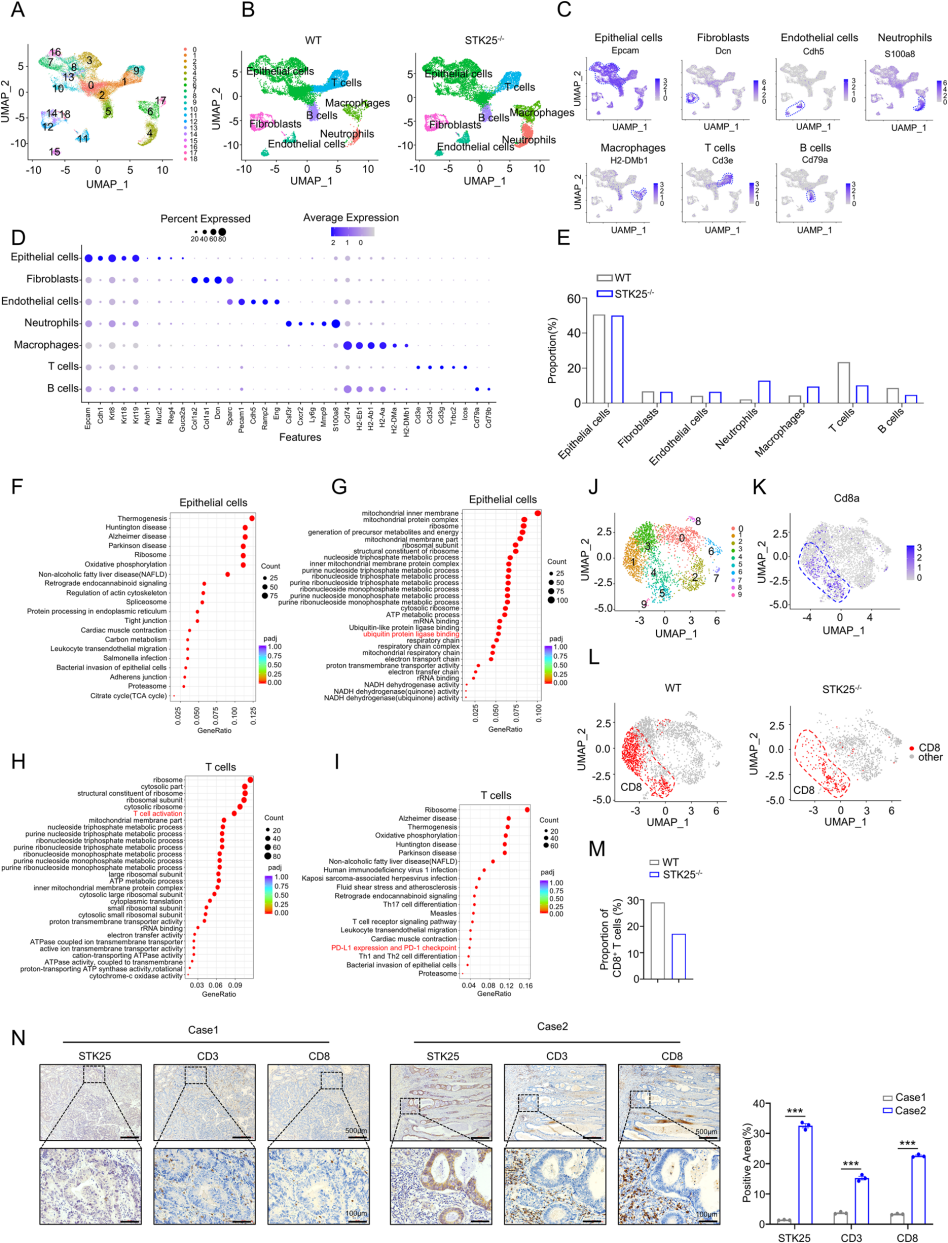
Whole‐body knockout of STK25 led to decreased infiltration of CD8^+^T cells in AOM/DSS‐induced CRC mouse models. A) UMAP plot of the analyzed single cells from mixed CRC samples of three individual wild‐type mice or STK25^−/−^ mice after AOM/DSS treatment. Each color represents one cluster. B) UMAP plot of cell subsets in WT and STK25^−/−^ mice treated with AOM/DSS. C) Feature plots illustrating the expression of known marker genes, including Epcam, Dcn, Cdh5, S100a8, H2‐DMb1, Cd3e, and Cd79a across different cell types. D) Dot plots illustrating expression patterns of known marker genes in the seven cell sub‐clusters. The color and size of individual dots represent the expression levels and cell fraction of the marker genes, respectively. E) The proportions of each cell type (E) in WT and STK25^−/−^ mice treated with AOM/DSS. F,G) GO (F) and KEGG (G) enrichment analyses for the differential expression genes (DEGs) between WT and STK25^−/−^ mice cancer cells. H,I) GO (H) and KEGG (I) enrichment analyses for the DEGs in intra‐tumoral T cells of WT and STK25^−/−^ mice. J) UMAP plot of ten T cell subsets in CRC of mice, with each cell color coded for cell clusters. K) UMAP analysis identified CD8^+^ T cells according to their marker gene Cd8a. L,M) Density plots of CD8^+^ T cells in T cells from WT and STK25^−/−^ mice (L) after AOM/DSS treatment. Corresponding bar graphs show quantification of CD8^+^ T cell percentage (M). N) Representative images of IHC staining for STK25, CD3, and CD8 in tumor tissues from CRC patients. Scale bars, 500 and 100 µm.

To investigate the underlying mechanism of STK25 in CRC, we performed single‐cell sequencing (scRNA‐seq) on intestinal tumors from STK25^−/−^ and WT mice. After particular quality control filtering, a total of 23822 cells (11327 and 12495 cells for control and STK25^−/−^ tumors, respectively) were obtained for subsequent analysis. Dimensional reduction and unsupervised clustering revealed 19 distinct cell clusters based on their expression profiles (**Figure** **2**A; Figure S2A, Supporting Information). According to the expression of canonical markers, all single cells were classified into seven major types, including epithelial cells, fibroblasts, endothelial cells, neutrophils, macrophages, T cells, and B cells (Figure 2B–D; Figure S2B–D, Supporting Information). Compared to the WT group, STK25^−/−^ tumors exhibited a higher percentage of endothelial cells, neutrophils, and macrophages, but a lower proportion of T cells and B cells (Figure 2E).

Subsequently, we analyzed how global STK25 depletion affects the transcriptional and functional state of cancer epithelial cells. To determine the genes regulated by STK25 in CRC cells, we performed the differential expression analysis on cancer cell transcriptomes from STK25^−/−^ and control samples. The top differential expression genes (DEGs) are shown in the volcano plot and heatmap (Figure S2E, Supporting Information). Based on the Kyoto Encyclopedia of Genes and Genomes (KEGG) as well as Gene Ontology (GO) analyses, STK25 might be involved in processes related to ribosome, oxidative phosphorylation, multiple ribonucleoside triphosphate metabolic process, and ubiquitin protein ligase binding (Figure 2F,G).

The immune cell populations in the tumor microenvironment (TME) are critical for modulating tumor progression and the efficacy of cancer immunotherapy.^[^ ^25^ ^]^ Since the percentage of T cells was significantly reduced in the STK25^−/−^ group (Figure 2E), we determined the functional state of T cells in the CRC microenvironment by analyzing T cells from both groups. Gene signature and pathway enrichment analyses were performed between the two groups. The top DEGs were displayed in the volcano plot and heatmap (Figure S2F, Supporting Information). Analysis of DEGs of T cells between the STK25^−/−^ tumors and the WT tumors showed a considerable enrichment in the ribosomal synthesis and oxidative phosphorylation pathway (Figure 2H,I). Interestingly, we also observed a significant enrichment in T cell activation and the PD‐1/PD‐L1 signaling pathway (Figure 2H,I), suggesting that STK25 deficiency‐induced tumor progression might be related with the activation status of T cells.

Cytotoxic CD8^+^ T cells are the predominant subset of activated T cells, responsible for exerting cell‐killing effects by directly recognizing and eliminating transformed cells.^[^ ^26^, ^27^ ^]^ To further characterize the functional state of T cells, we subclustered all 3913 T cells by using the unsupervised clustering analysis (Figure 2J). Subsequently, we annotated CD8^+^ T cells by examining Cd8a gene levels (Figure 2K). The results suggested that the proportion of CD8^+^ T cells was remarkably lower in the TME of STK25^−/−^ mice compared to wild‐type mice (Figure 2L,M). However, we observed an increased infiltration of CD4^+^ T cells in tumor tissues following STK25 knockout (Figure S2G,H, Supporting Information). Although CD4^+^ T cells primarily mediate anti‐tumor immunity, expansion of certain subsets may contribute to an immunosuppressive microenvironment. It is possible that the significant rise in regulatory T cell (Treg) proportion may account for this contradiction (Figure S2I–K, Supporting Information).

In tumor tissues from CRC patients, we observed that STK25‐low CRC patients had a lower proportion of CD3‐positive cells and CD8‐positive cells compared to the STK25‐high CRC patients by IHC staining (Figure 2N). These findings indicate that STK25 deficiency reduces T cell infiltration, particularly CD8^+^ T cells, and attenuates the anti‐tumor immune response.

### 2.3 STK25 Enhances the Cytotoxic Activity of T Cells In Vitro

**Figure 3:**
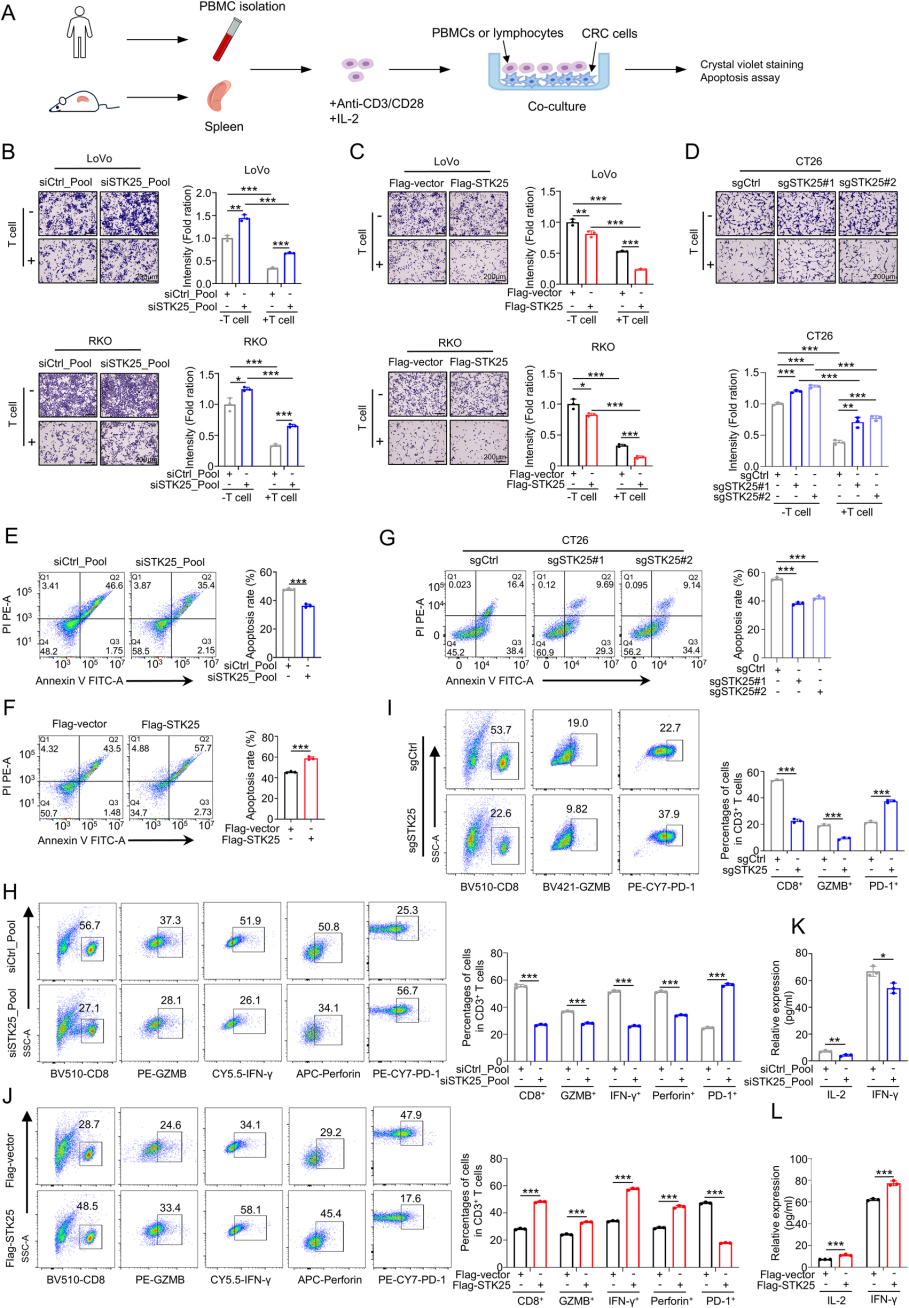
STK25 depletion in CRC cells suppresses T cell‐mediated cytotoxicity. A) Schematic illustration of a direct co‐culture of CRC cells and PBMCs. B–D) CRC cells transfected with pooled siRNA (B), plasmids (C), or sgRNA (D) were co‐cultured with activated PBMCs for 72 h, followed by crystal violet staining. Bar graphs show quantifications of the ratio of live cells. E–G) Apoptotic tumor cells detected by flow cytometry following co‐culture of PBMCs with siSTK25 (E), flag‐STK25 (F), or sgSTK25 (G) CRC cells. Apoptotic cells include both early and late apoptotic cells, as determined by Annexin V‐FITC and PI positivity. H–J) The functional status of activated PBMCs in each group was analyzed by flow cytometry. The plots and graphs showed percentages of CD8^+^ T cells, GZMB^+^ CD8^+^ T cells, IFN‐γ^+^ T cells, perforin^+^ cells, and CD8^+^ PD‐1^+^ T cells of PBMCs co‐cultured with siSTK25 (H), sgSTK25 (I), or flag‐STK25 (J) CRC cells. K,L) The levels of IL‐2 and IFN‐γ were detected in the supernatant of the co‐culture system containing PBMCs and STK25 knockdown (K) or overexpressing (L) CRC cells by ELISA assays. Results represent mean ± SD from three independent experimental replicates, analyzed by two‐way ANOVA. ^*^ *p* < 0.05, ^**^ *p* < 0.01, and ^***^ *p* < 0.001.

T cells are essential for tumor killing in vivo and in vitro. To assess whether the anti‐tumor ability of STK25 is dependent on T cells, CRC cells were co‐cultured with T cells to determine the impact of STK25 on T cell activity. First, we determined the depletion and overexpression efficiency of STK25 in CRC cells (Figure S3A–C, Supporting Information). Then we isolated and stimulated human and mouse T cells from peripheral blood and mouse splenocytes, respectively. LoVo and RKO cells were co‐cultured with human T cells, and CT26 cells were co‐cultured with mouse T cells (**Figure** **3**A). The results indicated that depletion of STK25 in tumor cells significantly prevented them from being killed by activated T cells, while overexpression of STK25 in tumor cells yielded the opposite effect (Figure 3B–D). Consistently, flow cytometry analysis of CRC cells collected from the co‐culture system revealed that silencing STK25 in tumor cells inhibited T cell‐mediated tumor cell killing, resulting in a reduced percentage of the apoptotic CRC cells. This suggests that STK25 deficiency in tumor cells suppresses T cell cytotoxicity (Figure 3E–G). These findings were also supported by LDH assay with consistent results (Figure S3D, Supporting Information).

Furthermore, co‐culture with STK25‐depleted CRC cells significantly decreased the percentage of activated CD8^+^T cells characterized by granzyme B, IFN‐γ, and perforin positive ratios, and increased the percentage of exhausted CD8^+^ T cells marked by PD‐1 and Tim3 (Figure 3H,I; Figure S3E, Supporting Information). In contrast, STK25 overexpression promoted T cell activation (Figure 3J). This was further validated by measuring the levels of IL‐2 and IFN‐γ in supernatants and T cells from co‐cultures by ELISA and RT‐qPCR, respectively (Figure 3K,L; Figure S3F,G, Supporting Information). Collectively, STK25 stimulates the release of killer factors, suggesting that STK25 is capable of enhancing T cell cytotoxicity by inducing T cell activation.

### 2.4 STK25 Facilitates T Cell‐Mediated Killing Efficacy in a PD‐1/PD‐L1 Signaling‐Dependent Manner

**Figure 4:**
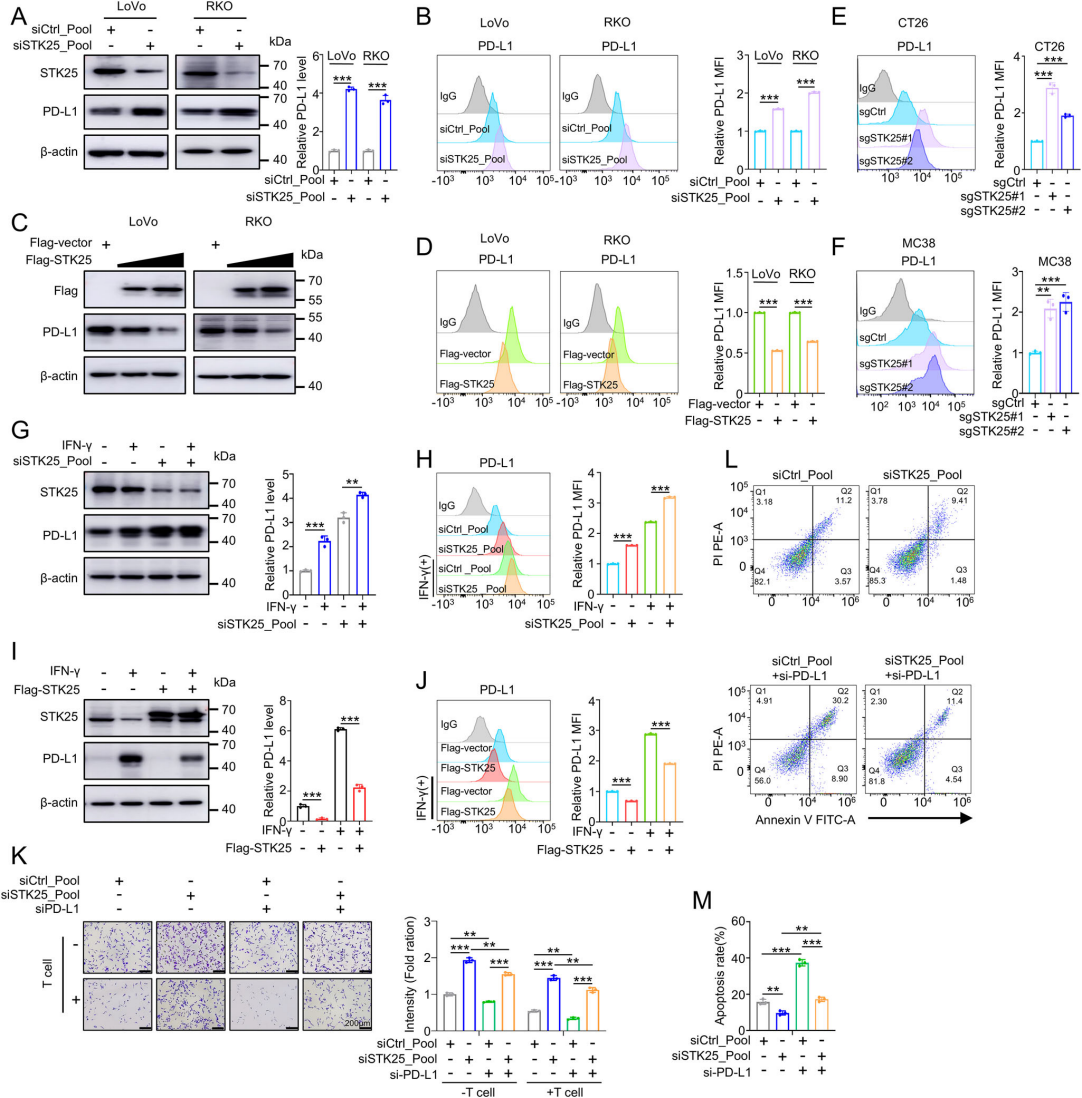
STK25 deficiency inhibits the cytotoxic capacity of T cells by upregulating PD‐L1. A,B) Western blot (A) and flow cytometry analysis (B) of PD‐L1 levels on CRC cell lines transfected with pooled siSTK25 or control siRNA. Bar graphs show the gray value analysis and the mean fluorescence intensity (MFI). C,D) The expression of PD‐L1 in STK25 overexpressing CRC cells was analyzed by Western blot (C) and flow cytometry analysis (D). E,F) The surface levels of PD‐L1 were assessed in CT26 (E) and MC38 (F) cells transfected with sgSTK25 or control sgRNA. G–J) Western blot and flow cytometric analysis showing the effect of STK25 depletion (G,H) or overexpression (I,J) on IFN‐γ‐mediated PD‐L1 expression in the indicated cells. Bar graphs show the results of quantitation. K–M) Activated PBMCs were co‐cultured with RKO cells transfected with indicated siRNAs. Viable tumor cells were stained by crystal violet (K). Scale bar, 200 µm. The normalized ratio of live tumor cells is shown in the bar graph. Apoptotic tumor cells were examined through flow cytometry (L) and presented with bar graphs (M). The above experiments were repeated three times independently. The data are presented as the mean ± SD, and P values were calculated using an unpaired two‐sided Student's *t*‐test. ^**^ *p* < 0.01 and ^***^ *p* < 0.001.

Based on the scRNA‐seq data indicating a correlation between STK25 and the PD‐1/PD‐L1 signaling pathway (Figure 2I), a crucial immune checkpoint, we sought to investigate whether STK25‐mediated alterations in T cell numbers and function were linked to PD‐1/PD‐L1 signaling pahway. Western blot and flow cytometry analysis were employed to assess the expression of PD‐L1 in CRC cells with silencing or overexpressing STK25. The results demonstrated a notable elevation in the levels of total PD‐L1 and membrane PD‐L1 in siSTK25 cells in comparison to control cells (**Figure** **4**A,B). Moreover, a gradual reduction in PD‐L1 levels was observed following STK25 dose‐dependent transfection (Figure 4C,D; Figure S4A, Supporting Information). Similarly, the levels of PD‐L1 increased in STK25‐depleted mouse tumor cells, including CT26 and MC38 cells (Figure 4E,F). These results suggest that STK25 could negatively regulate PD‐L1 expression. In addition to PD‐L1, we examined the association between STK25 and other immune checkpoint‐related genes, including PD‐L2, PVR, and Siglec15. The results showed that neither knockout nor overexpression of STK25 affected the mRNA or protein expression levels of these genes in CRC cells (Figure S4B–F, Supporting Information).

Since previous studies have demonstrated that IFN‐γ is one of the most potent inducers of PD‐L1,^[^ ^28^, ^29^ ^]^ we proceeded to examine whether STK25 could regulate the induction of PD‐L1 stimulated by IFN‐γ. The results showed that the knockdown of STK25 markedly augmented the upregulation of PD‐L1 by IFN‐γ (Figure 4G,H), whereas the overexpression of STK25 suppressed IFN‐γ‐induced PD‐L1 expression (Figure 4I,J). These findings suggest that STK25 plays a critical role in the IFN‐γ‐induced modulation of PD‐L1 in CRC cells.

Subsequently, in order to further investigate whether the STK25‐mediated T cell function depend on PD‐L1 levels, we knocked down PD‐L1 to block the pathway in STK25‐depleted CRC cells (Figure S4G,H, Supporting Information). T cell‐mediated killing assay and flow cytometry were performed in CRC cells co‐cultured with activated T cells. The results showed that knockdown of STK25 inhibited T cell function, and knockdown of PD‐L1 rescued this effect (Figure 4K–M). Overall, these findings show that STK25 deficiency‐induced T cell dysfunction is dependent on the PD‐L1 levels in CRC.

### 2.5 STK25 Phosphorylates PD‐L1 and Attenuates PD‐L1 Protein Stability via the Ubiquitin‐Proteasome Pathway

**Figure 5:**
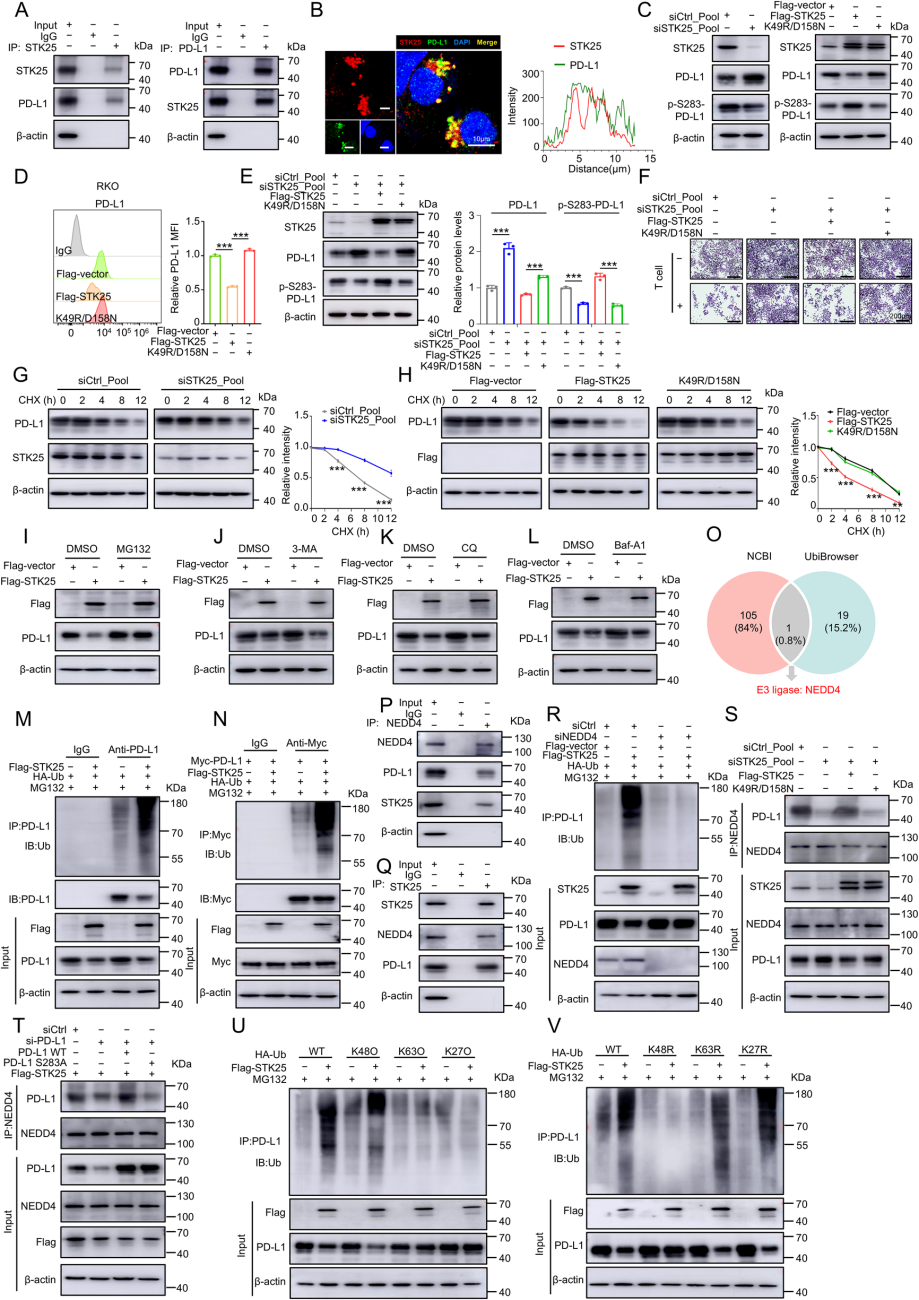
STK25‐mediated phosphorylation of PD‐L1 triggers its degradation through the ubiquitin‐proteasome pathway. A) STK25 interacts with PD‐L1 in RKO cells. Co‐immunoprecipitation (Co‐IP) was performed using an anti‐STK25 antibody (left) or anti‐PD‐L1 antibody (right). B) Colocalization of endogenous STK25 (red) and PD‐L1 (green) in RKO cells. The fluorescence intensity graph (bottom) shows the level of co‐localization. Scale bar, 10 µm. C) The phosphorylation of PD‐L1 at Ser283 was examined by Western blot analysis in STK25 knockdown (left) or overexpression (right) CRC cells. D) PD‐L1 expression on the cell surface was examined using flow cytometry in CRC cells transfected with STK25 plasmids carrying full‐length STK25 cDNA (WT) or carrying the K49R/D158N mutation. E) Levels of total and phosphorylated (Ser 283) PD‐L1 were measured in STK25 knockout CRC cell lines transfected with indicated plasmid. F) STK25 knockdown CRC cells transfected with STK25 WT or K49R/D158N mutation plasmids were co‐cultured with activated PBMCs for 72 h and then subjected to crystal violet staining. Scale bar, 200 µm. G,H) Western blot analysis showed the PD‐L1 protein half‐life in STK25 knockdown (G) or overexpression (H) CRC cells exposed to CHX for the indicated time period. I–L) CRC cells transfected with the Flag‐STK25 plasmid were treated with MG132 (I), 3‐MA (J), CQ (K) or Baf‐A1 (L). The proteins were then detected via Western blot analysis. M,N) RKO cells (M) or HEK293T cells (N) were transfected with the indicated constructs. Ubiquitinated endogenous (M) and exogenous (N) PD‐L1 were immunoprecipitated and analyzed by Western blot using anti‐ubiquitin antibody. Prior to the ubiquitination analysis, MG132 was administered to the cells. O) Venn diagram of overlapping E3 ubiquitin ligases of PD‐L1 predicted to interact with STK25 from online databases (NCBI and UbiBrowser). P,Q) Endogenous STK25, NEDD4, and PD‐L1 are present in the same complex. Co‐IP was performed using an anti‐NEDD4 antibody (P) or anti‐STK25 antibody (Q). R) Immunoprecipitation (IP) analysis of the ubiquitination of PD‐L1 in WT and NEDD4 knockdown HEK293T cells transfected with the indicated constructs. MG132 was applied to cells for 12 h prior to harvest. S,T) Western blot analysis of anti‐NEDD4 IP derived from RKO cells transfected with indicated plasmids or siRNAs. U,V) Ubiquitination assays were performed using exogenously overexpressed Flag‐STK25 and HA‐Ub (WT/K48O/K63O/K27O/K48R/K63R/K27R) in HEK293T cells. Cells were treated with MG132 after 48 h of transfection, and IP was performed to analyze the ubiquitination of PD‐L1. Ub (K48O), ubiquitin with all lysines mutated to arginine except K48. Ub (K48R), ubiquitin with only K48 mutated to arginine. Data represent mean ± SD from three independent experiments, analyzed by unpaired two‐tailed Student's t‐test. **p < 0.001 and ^***^ *p* < 0.001.

Given that STK25 has been demonstrated to inhibit PD‐L1 levels and that STK25 is a serine/threonine protein kinase, we postulated that STK25 phosphorylates PD‐L1 to regulate its stability. The initial step was to ascertain whether there was a physical interaction between STK25 and PD‐L1 through reciprocal co‐immunoprecipitation (co‐IP). The result revealed an endogenous association between STK25 and PD‐L1 (**Figure** **5**A). Concordantly, co‐localization of STK25 with PD‐L1 was observed in cancer cells (Figure 5B; Figure S5A, Supporting Information).

We then performed Western blot to assess the phosphorylation of PD‐L1 in CRC cells with knockdown or overexpression of STK25. The results showed that silencing STK25 could downregulate the PD‐L1 phosphorylation at Ser283 (Figure 5C), a known phosphorylation site that could trigger PD‐L1 degradation.^[^ ^30^ ^]^ Moreover, overexpression of the wild‐type STK25 was capable of enhancing PD‐L1 phosphorylation at Ser283 and inhibiting PD‐L1 levels. Conversely, the kinase‐dead STK25 (K49R/D158N) failed to elicit these effects (Figure 5C; Figure S5B, Supporting Information). Similarly, the flow cytometry analysis also demonstrated that the kinase‐dead STK25 had no effect on PD‐L1 membrane protein levels (Figure 5D).

Furthermore, rescue experiments were performed by overexpressing either WT or the kinase‐dead form (K49R/D158N) of STK25 in STK25‐silenced CRC cells. The results showed that only the WT STK25 was capable of reversing the effect of STK25 knockdown on PD‐L1 levels and tumor cell proliferation (Figure 5E,F). These findings suggest that the kinase activity of STK25 is indispensable for the regulation of PD‐L1 phosphorylation and protein levels.

Previous research has suggested that the PD‐L1 phosphorylation at Ser283 reduces the half‐time of the PD‐L1 protein.^[^ ^31^ ^]^ We therefore aimed to ascertain whether STK25 contributes to PD‐L1 stabilization and degradation. The results of a cycloheximide (CHX) chase experiment showed that STK25 deficiency enhanced the stability of PD‐L1 protein in CRC cells exposed to CHX (Figure 5G). Conversely, the overexpressing wild‐type STK25 could hasten the PD‐L1 degradation, while the kinase‐dead STK25 (K49R/D158N) had no appreciable impact on the half‐life of PD‐L1 (Figure 5H).

Subsequently, to investigate the underlying mechanisms by which STK25 regulates the degradation of PD‐L1, we treated the cells with proteasome inhibitor (MG132), autophagosome formation inhibitor 3‐MA, lysosomal inhibitor CQ, or autophagosome‐lysosome fusion inhibitor Baf‐A1. The results showed that only MG132, but not 3‐MA, CQ, or Baf‐A1, could rescue the decreased level of PD‐L1 protein induced by STK25 overexpression (Figure 5I–L; Figure S5C–F, Supporting Information). The stabilizing impact of STK25 on PD‐L1 did not further raise the PD‐L1 protein level when MG132 was present (Figure S5G, Supporting Information). These data suggested that STK25 promotes PD‐L1 degradation through the proteasomal pathway.

Furthermore, the results of the ubiquitination‐based IP assay revealed that STK25 could elevate the ubiquitination levels of endogenous and exogenous PD‐L1 in HEK293T cells (Figure 5M,N). Therefore, these findings indicate that STK25 interacts with PD‐L1, promoting its phosphorylation and subsequent degradation via the ubiquitin‐proteasome pathway.

To identify the regulatory factor acting as a scaffold between STK25 and PD‐L1, we first analyzed the protein–protein interaction (PPI) between STK25 and common tumor‐related proteins using the NCBI database (Table S1, Supporting Information). Subsequently, an intersection was extracted between these STK25‐interacting proteins and PD‐L1‐associated E3 ubiquitin protein ligases provided from the UbiBrowser database (Figure 5O; Table S1, Supporting Information). Interestingly, we found that NEDD4 might be the most likely regulator involved in STK25‐mediated regulation of PD‐L1 expression. NEDD4 is an E3 ubiquitin ligase that facilitates target protein ubiquitination and degradation by physically interacting with protein substrates, including PD‐L1.^[^ ^32^ ^]^ As anticipated, our results showed the interaction between endogenous NEDD4 and PD‐L1 (Figure 5P; Figure S5H, Supporting Information). Meanwhile, co‐IP assays demonstrated the binding between endogenous STK25 and NEDD4 (Figure 5P,Q), suggesting the formation of a tripartite complex involving STK25, NEDD4, and PD‐L1. Moreover, NEDD4 deficiency increased the PD‐L1 protein level in CRC cells, while STK25 did not appear to affect NEDD4 expression levels (Figure S5I,J, Supporting Information). Furthermore, to explore whether the STK25‐mediated PD‐L1 ubiquitination depends on NEDD4, we assessed the ubiquitination level of PD‐L1 in NEDD4‐silenced cells. We found that STK25‐mediated PD‐L1 ubiquitination was completely abolished upon NEDD4 knockdown (Figure 5R). Collectively, our findings suggest that STK25 may regulate PD‐L1 ubiquitination by facilitating the recruitment of the NEDD4 ubiquitin ligase to PD‐L1.

To determine the role of STK25 in the interaction between NEDD4 and PD‐L1, we performed rescue experiments in STK25‐knockdown CRC cells. Results demonstrated that STK25 silencing significantly impaired the binding of NEDD4 to PD‐L1 (Figure 5S). Re‐expression of exogenous wild‐type STK25 in the silenced cells rescued this interaction, whereas expression of a kinase‐dead STK25 (K49R/D158N) mutant failed to restore it (Figure 5S). These findings indicate that STK25 and its functional kinase activity are crucial for the NEDD4‐PD‐L1 interaction.

To further test whether STK25‐mediated phosphorylation of PD‐L1 at Ser283 affects the interaction between PD‐L1 and NEDD4, we overexpressed wild‐type PD‐L1 or phosphorylation null mutant PD‐L1 (S283A) in PD‐L1 knockdown CRC cells. Compared with wild‐type PD‐L1, the PD‐L1 S283A exhibited a reduced interaction with NEDD4 (Figure 5T), suggesting that the phosphorylation status of PD‐L1 at Ser283 facilitates its binding to NEDD4. Subsequent ubiquitination assays further demonstrated that phosphorylation of PD‐L1 at Ser283 enhances NEDD4‐mediated ubiquitination of PD‐L1 compared to the S283A mutant (Figure S5K, Supporting Information). Overall, these data suggest that STK25 kinase‐dependent phosphorylation of PD‐L1 at Ser283 facilitates NEDD4 binding, thereby promoting NEDD4‐mediated ubiquitination and degradation of PD‐L1.

Previous study demonstrated that NEDD4 promotes K48‐linked polyubiquitination of PD‐L1, leading to its proteasomal degradation.^[^ ^32^ ^]^ Consistently, our results showed that STK25‐mediated PD‐L1 ubiquitination was only detectable with K48O‐Ub plasmid (ubiquitin with only the Lys48 residue intact), but not with K63O‐Ub or K27O‐Ub plasmids (Figure 5U). Furthermore, the K48R (mutation of Lys48 to Arg) mutant completely abolished STK25‐induced PD‐L1 ubiquitination. In contrast, STK25 retained its ability to promote PD‐L1 ubiquitination when K63R and K27R mutants were used (Figure 5V). Taken together, these findings indicate that STK25 regulates NEDD4‐mediated PD‐L1 K48‐linked ubiquitination and protein levels.

### 2.6 STK25 Depletion Enhances the Efficacy of Checkpoint Blockade Therapy In Vivo

**Figure 6:**
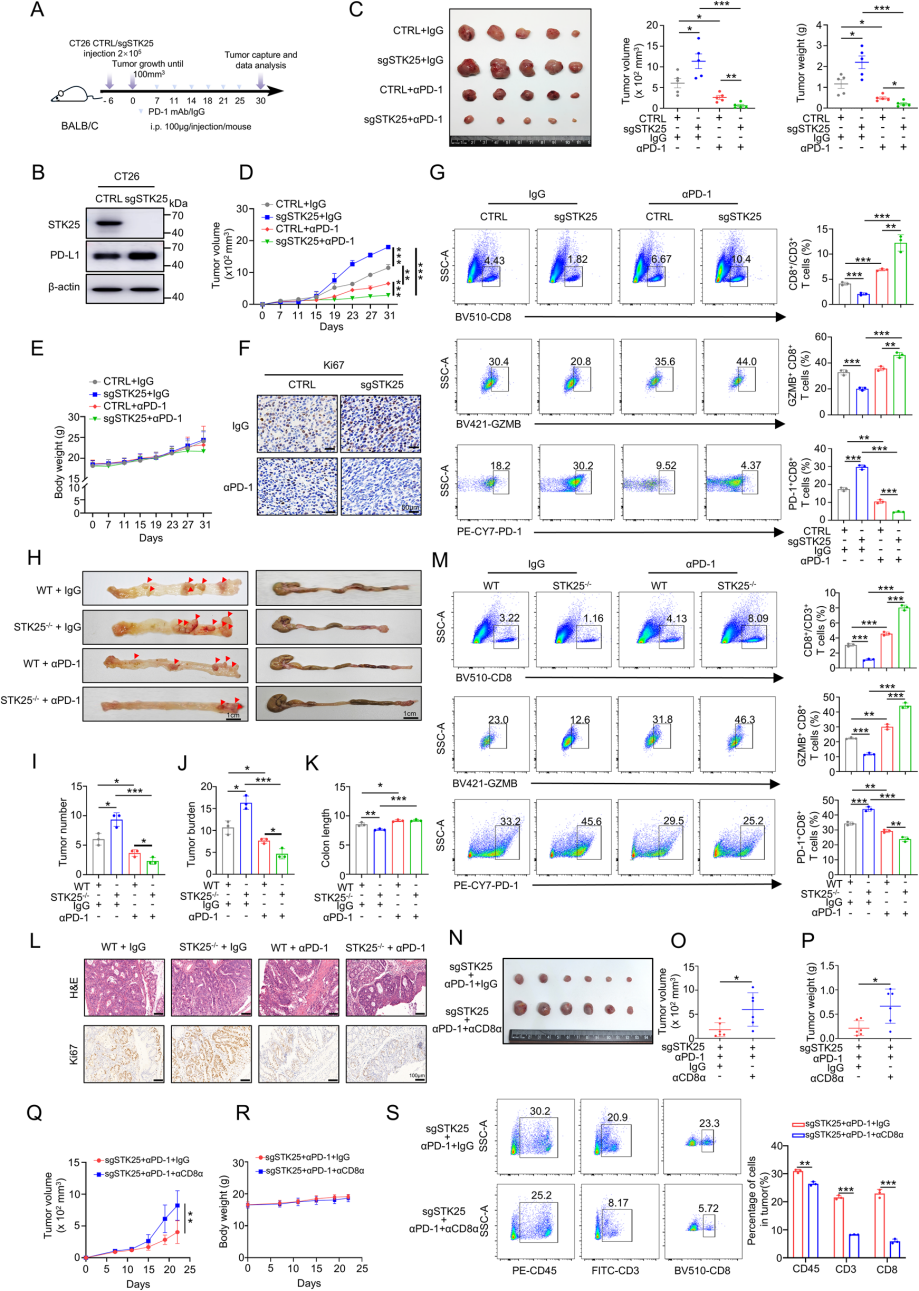
STK25 depletion combined with ICB therapy significantly inhibits CRC growth. A) Diagrammatic illustration of the dosing schedule and xenograft mouse model. PD‐1 mAb treatment was administered to mice with either control or sgSTK25 CT26 tumors. B) STK25 and PD‐L1 protein expression in CT26 cells determined by Western blot. C) Tumor images of the mice in different groups (n = 5 per group). The tumor volume and tumor weight were quantified and shown in bar graphs. D,E) Tumor growth curves (D) and body weights (E) of mice were measured every 4 days. F) Representative images of Ki67 staining of tumor xenograft tissues from mice in different groups. Scale bar, 50 µm. G) The proportions of infiltrating CD8^+^ T cells, Granzyme B^+^ T cells, and PD‐1^+^ CD8^+^ T cells of murine tumor tissues (n = 3 per group) were detected using flow cytometry. H–K) Representative morphological images of the opened and completed colons from WT and STK25^−/−^ mice treated with IgG isotype control or PD‐1 mAb (H) (n = 3 per group). The tumor volume (I), tumor burden (J), and colon length (K) were quantified and shown in bar graphs. L) Representative photographs of H&E and Ki67 staining of murine tumor tissues from WT and STK25^−/−^ mice with PD‐1 mAb therapy. Scale bar, 100 µm. M) The flow analysis shows the proportions of infiltrating CD8^+^ T cells, Granzyme B^+^ T cells, and PD‐1^+^ CD8^+^ T cells in mice tumor tissues from WT and STK25^−/−^ mice received IgG isotype control or PD‐1 mAb (n = 3 per group). N–S) BALB/C mice were implanted with sgSTK25 CT26 cells. PD‐1 mAb (αPD‐1) or CD8α mAb (αCD8α) were administered to mice. (N) Tumor images of the mice in different groups (n = 6 per group). (O, P) The tumor volume(O) and tumor weight (P) were quantified and shown in plots. (Q, R) Tumor growth curves (Q) and body weights (R) of mice were measured every 5 days. (S) The proportions of infiltrating CD3^+^ T cells, CD8^+^ T cells in CD45^+^ cells of murine tumor tissues (n = 3 per group) were detected using flow cytometry. Results represent mean ± SD from three independent experimental replicates, analyzed by two‐way ANOVA. ^*^ *p <* 0.05, ^**^ *p* < 0.01, and ^***^ *p* < 0.001.

Given the reduced infiltration of CD8^+^ T cells and the suppressed microenvironment in CRC of STK25^−/−^ mice (Figure 2N–P), as along with the established role of STK25 in regulating the PD‐1/PD‐L1 pathway, we postulated that STK25 may influence the efficacy of checkpoint blockade therapy. To test the hypothesis, we generate subcutaneous xenografts using CT26 cells in immunocompetent BALB/c mice that were treated with either IgG isotype control (IgG2a) or PD‐1 mAb (**Figure** **6**A). The expression of STK25 and PD‐L1 in the CT26 cells was validated by Western blot (Figure 6B). The loss of STK25 resulted in a significant increase in tumor growth compared to the control mice (Figure 6C,D), which is consistent with the results observed in STK25^−/−^ mice. Moreover, it is noteworthy that the STK25 knockout and PD‐1 mAb combined treatment group exhibited the most pronounced reduction in tumor burden compared with the other groups (Figure 6C,D), without significant body weight loss (Figure 6E). In addition, Ki67 staining by IHC revealed that combined STK25 deficiency and PD‐1 mAb treatment resulted in extensive tumor cell death and a significant reduction in the proliferation capacity (Figure 6F). This intriguing result suggested that STK25 deficiency renders CT26 cells more susceptible to PD‐1 mAb therapy.

Moreover, we investigated the alteration of the CD8^+^ T cells in murine CRC tumors in response to PD‐1 mAb treatment. Flow cytometry analysis showed that the loss of STK25 caused a significant decrease in the number of CD8^+^ T cells and GZMB^+^ CD8^+^ T cells, while there was an increase in the number of PD‐1^+^ CD8^+^ T cells (Figure 6G; Figure S6A, Supporting Information). Of note, in STK25 knockout groups, we were surprised to find that PD‐1 mAb led to the most notable increase in the percentage of CD8^+^ T cells and GZMB^+^ CD8^+^ T cells, along with a decrease in the proportion of PD‐1^+^ CD8^+^ T cells (Figure 6G). These findings suggest that STK25 deficiency impairs CD8^+^ T cell‐induced antitumor immunity, thereby facilitating tumor immune evasion. However, the diminished T‐cell activation resulting from STK25 depletion augmented the efficacy of anti‐PD‐1 treatment. Furthermore, IHC profiling of tumor‐infiltrating immune subsets revealed no significant changes in the proportions of dendritic cells (DCs, CD11c), myeloid‐derived suppressor cells (MDSCs, Ly6G), macrophages (CD68), and regulatory T cells (Tregs, Foxp3) following combination treatment (Figure S6B, Supporting Information). These data indicate that STK25‐mediated PD‐L1 regulatory axis predominantly modulates CD8^+^ T cell function rather than myeloid or regulatory immune cell populations.We next investigated whether STK25 depletion enhances the antitumor effects of PD‐1 blockade in a more preclinically relevant CRC model. The WT and STK25^−/−^ mice were treated with AOM/DSS to induce CRC and then administered with the IgG isotype or PD‐1 mAb. Similar to the results observed in the subcutaneous mouse model, we found that the STK25^−/−^ mice were more susceptible to PD‐1 mAb therapy, as confirmed by reduced tumor burden and increased colon length (Figure 6H–K). Consistently, Staining with H&E and IHC analysis of Ki‐67 confirmed that tumor cell proliferation was significantly decreased in STK25^−/−^ mice treated with PD‐1 mAb (Figure 6L). Furthermore, compared with the control group, the infiltration of CD8^+^ T cells and the cytotoxicity indicator GZMB^+^ CD8^+^ T cells were increased, and the exhaustion indicator PD‐1^+^ CD8^+^ T cells were decreased in STK25^−/−^ mice with PD‐1 mAb treatment (Figure 6M). Taken that, our data indicated that loss of STK25 could enhance the effects of PD‐1 blockade on CRC tumors in the mouse model.

Moreover, CD8α mAb (αCD8α) was used to deplete CD8^+^ T cells in vivo to further determine whether CD8^+^ T cells are necessary for the synergistic effect of the combination treatment of STK25 depletion and PD‐1 mAb. The results showed that αCD8α cotreatment significantly diminished the therapeutic effects of the combination treatment with STK25 knockout and PD‐1 mAb by eliminating CD8^+^ T cells in the CRC mouse model (Figure 6N–R). Meanwhile, the percentages of CD8^+^ T cells in tumor tissues and spleens were significantly reduced after administration of αCD8α (Figure 6S; Figure S6C, Supporting Information). Together, these findings suggest that CD8^+^ T cells are required for the combined anti‐tumor efficacy of PD‐1 mAb and STK25 deficiency.

### 2.7 STK25‐PD‐L1 Axis Correlates with Immune Checkpoint Therapy Response in CRC Patients

**Figure 7:**
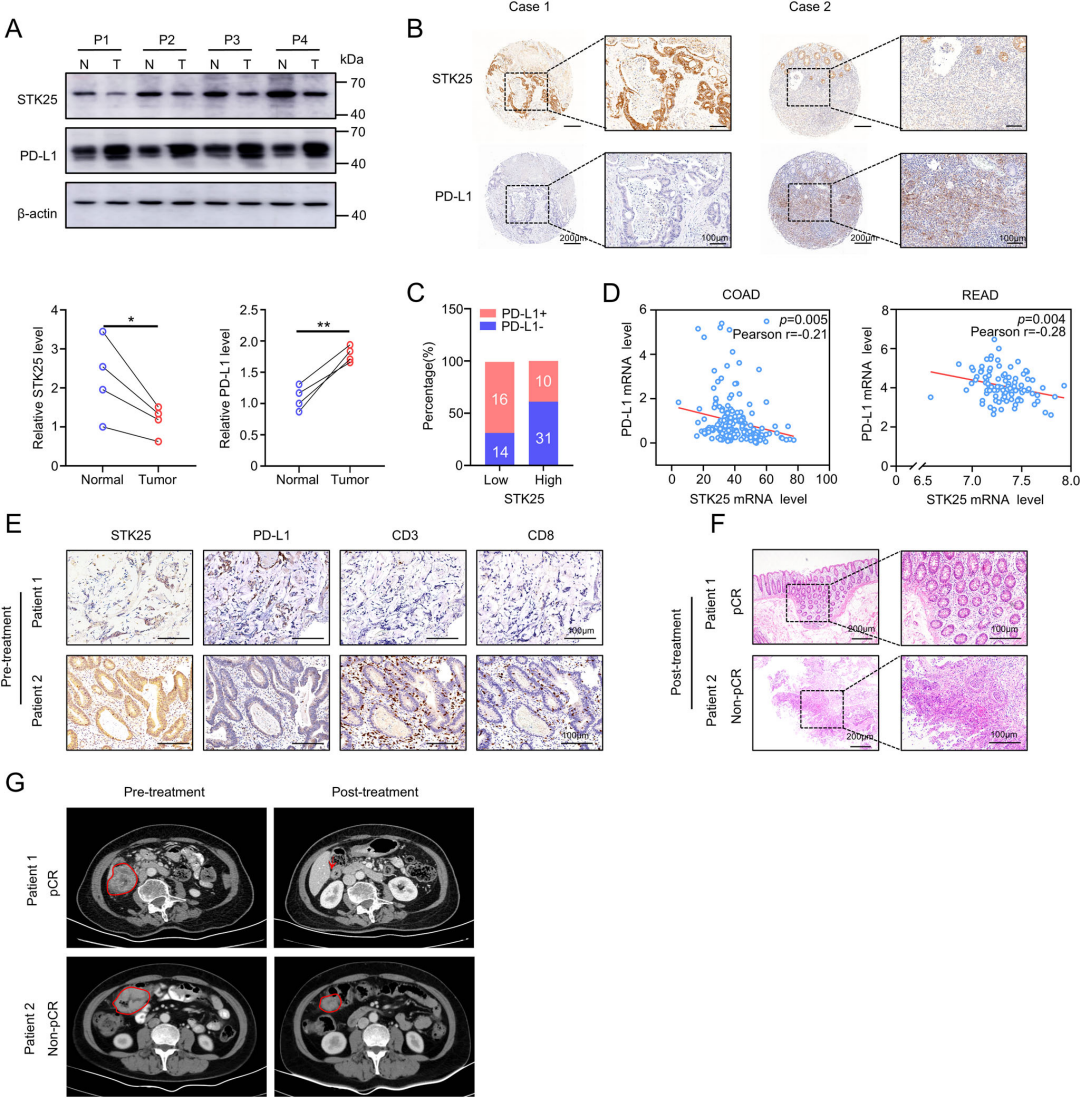
CRC patients with low STK25 expression show heightened sensitivity to ICB therapy. A)The protein levels of STK25 and PD‐L1 in CRC tissues and normal paired tissues were measured by Western blot analysis. Scatter plots show the quantification of band intensity. B) Representative images of STK25 and PD‐L1 staining in CRC tissues (n = 71). Scale bars, 200 and 100 µm. C) The stacked bar chart shows the percentage of PD‐L1‐negative and PD‐L1‐positive patients in the low and high STK25 CRC groups, respectively. Quantification of STK25 and PD‐L1 expression according to IHC scores in tumor tissues. D) Correlations between STK25 and PD‐L1 in colon adenocarcinoma (COAD) and rectal adenocarcinoma (READ) were calculated using the cBioPortal tool. E) The preoperative CRC patients treated with ICB therapy were analyzed. IHC staining using antibodies specific for STK25, PD‐L1, CD3, and CD8 in CRC tissues. Scale bar, 100 µm. F) H&E staining was conducted to identify the pathological complete responders (pCR) and non‐complete responders (non‐pCR) in postoperative patients. Scale bar, 200 and 100 µm. G) The location of the tumors according to the CT imaging were indicated with red circles. As shown by the red arrow, the tumor has completely disappeared. Statistical significance was assessed using two‐tailed Student's *t*‐tests. ^*^ *p* < 0.05 and ^**^ *p* < 0.01.

To demonstrate the clinical relevance of our findings, we first assessed the expression of STK25 and PD‐L1 in tumors or paracancerous tissues of CRC patients. Western blot analysis revealed significantly higher STK25 expression and lower PD‐L1 expression in the paracancerous tissues in compared to the tumor tissues, with STK25 levels showing a negative correlation with PD‐L1 expression (**Figure** **7**A; Table S2, Supporting Information). Next, we used a tissue microarray comprising 71 tumor samples from CRC patients paired with comprehensive clinical and pathologic details, to evaluate the expression of STK25 and PD‐L1. Likewise, the negative correlation between STK25 and PD‐L1 was validated (Figure 7B,C; Table S3, Supporting Information). Consistently, bioinformatics analysis of the CRC database from the TCGA showed a notable negative association of mRNA levels between STK25 and PD‐L1 (Figure 7D). Collectively, these findings highlight the clinical relevance of the STK25‐PD‐L1 axis in human CRC.

Next, we determined the association between the STK25‐PD‐L1 axis and the efficacy of ICB therapy in CRC patients. We recruited CRC patients undergoing ICB treatment and measured the levels of STK25, PD‐L1, CD3, and CD8 in CRC tissues obtained via colonoscopy prior to the initiation of ICB therapy (Figure 7E; Table S4, Supporting Information). According to the postoperative pathological results, we observed that CRC patients with low STK25 and high PD‐L1 levels had a favorable response to ICB therapy, and were classified as pathological complete remission (pCR) (Figure 7F). In contrast, patients with high STK25 and low PD‐L1 levels still had residual lesions after ICB treatment, and were classified as non‐pCR (Figure 7F). The results of CT imaging also confirmed this finding, as patients with low STK25 levels showed complete tumor disappearance following treatment (Figure 7G).

It is noteworthy that according to TCGA database, the negative correlation between STK25 and PD‐L1 was widely observed in a range of cancer types, including prostate adenocarcinoma (PRAD), liver hepatocellular carcinoma (LIHC), cervical squamous cell carcinoma and endocervical adenocarcinoma (CESC), and kidney renal clear cell carcinoma (KIRC) (Figure S7A, Supporting Information). Additionally, sequencing data from relevant clinical studies show that melanoma (PRJEB23709) or NSCLC (GSE126044) patients with low STK25 and high PD‐L1 expression were more likely to respond to ICB therapy, as indicated by the TIGER database (Figure S7B, Supporting Information). These findings suggest that the association between the STK25‐PD‐L1 axis and immune responses may be applicable to a varietyof cancers.

Collectively, these results confirmed that tumor patients with low STK25 and high PD‐L1 expression may potentially benefit from ICB therapy.

## 3 Discussion

**Figure 8:**
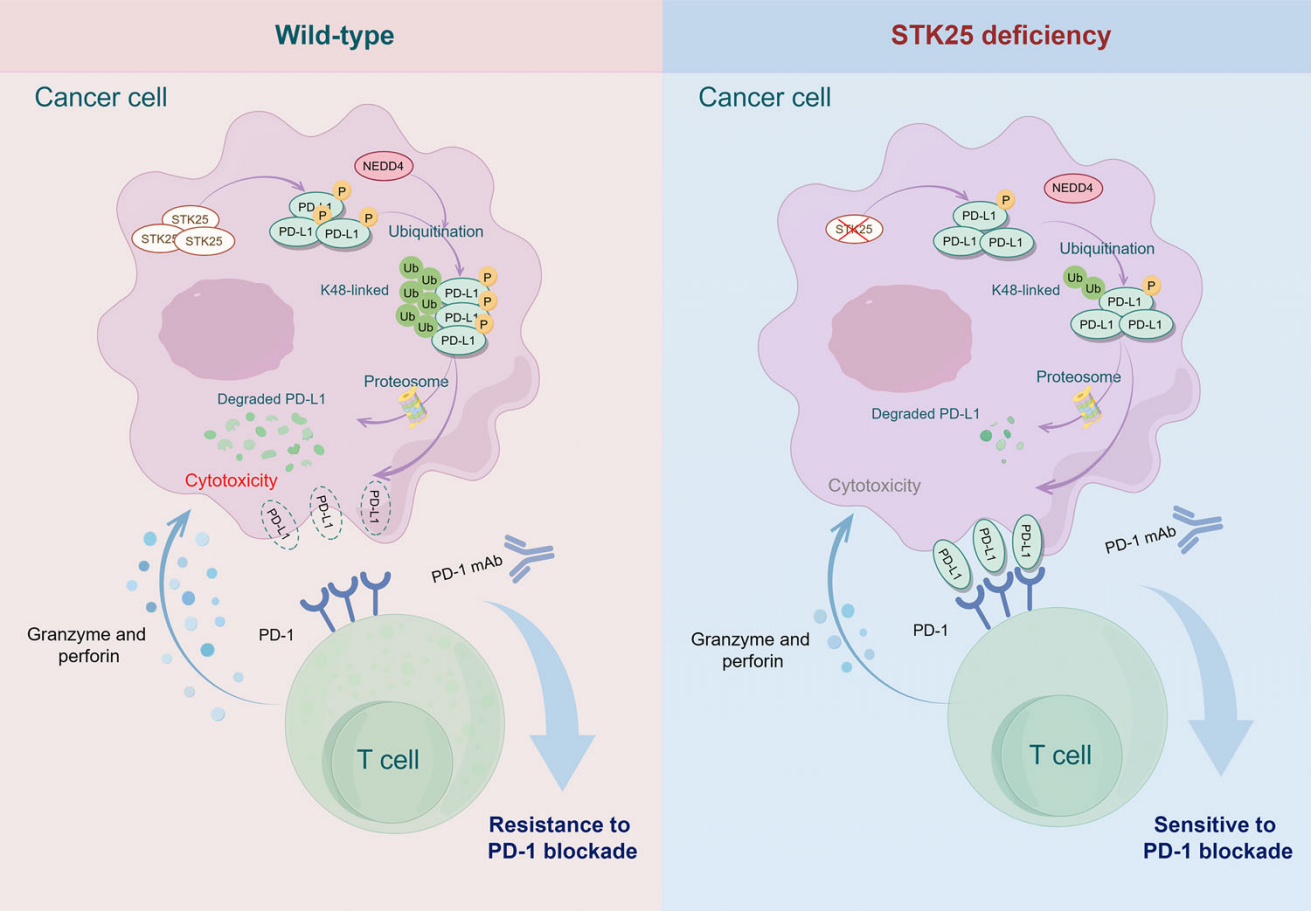
A schematic diagram depicts the potential mechanisms by which STK25 depletion promotes CRC immune escape. STK25 depletion upregulates the PD‐L1 levels by inhibiting the PD‐L1 phosphorylation‐mediated degradation, a process triggered by K48‐linked ubiquitination of PD‐L1 via NEDD4. Moreover, STK25 deficiency renders CRC tumors more sensitive to checkpoint blockade therapy. Created with Figdraw.com.

Although ICB therapies have achieved great success, the clinical benefits for CRC patients remain limited.^[^ ^33^ ^]^ Thus, it is imperative to explore the underlying mechanisms of immune escape in CRC and develop novel therapeutic approaches. In this study, we found that STK25 deficiency promoted CRC immune escape and tumor progression by inhibiting PD‐L1 phosphorylation and stabilizing PD‐L1 protein (**Figure** **8**). STK25 deficiency rendered CRC tumors more responsive to checkpoint blockade therapy. Moreover, we observed that CRC patients with low STK25 and high PD‐L1 expression exhibited a superior therapeutic outcome in CRC patients following ICB treatment. In summary, the current study identified STK25 as a key regulator of PD‐L1 degradation and demonstrated its potential as a therapeutic target to enhance ICB therapy in CRC.

The function of STK25 in cancer remains ambiguous. STK25 served as an oncogenic gene in fibrosarcoma and hepatocellular carcinoma but as a tumor suppressor in CRC, neuroblastoma, and breast cancer.^[^ ^34^, ^35^, ^36^, ^37^ ^]^ This discrepancy may be related to tumor types and the distinct mechanisms of action. Our previous studies found that STK25 attenuated the proliferation of CRC cells through modulation of glycolysis and cellular autophagy.^[^ ^22^, ^23^ ^]^ In this study, we found that STK25 depletion enhanced AOM/DSS‐induced colon tumorigenesis in the STK25^−/−^ mice. Moreover, we identified for the first time via scRNA‐seq that the immune microenvironment, especially the infiltration of CD8^+^ T cells, exerted an important role in STK25‐mediated tumor progression in CRC.

As a crucial mechanism influencing the efficacy of immunotherapy, PD‐L1 expression regulation has been extensively studied. Accumulating evidence indicates that cancer cells employ comprehensive mechanisms to modulate PD‐L1 levels. These regulatory processes can occur at both the genomic and epigenetic levels, involving gene amplification, structural variations, DNA methylation, histone methylation, histone acetylation, etc.^[^ ^38^, ^39^, ^40^, ^41^, ^42^ ^]^

In terms of the post‐translational modifications, phosphorylation, glycosylation, ubiquitination, and palmitoylation could influence PD‐L1 levels in cancer cells by affecting its stability.^[^ ^43^, ^44^, ^45^, ^46^ ^]^ The phosphorylation of PD‐L1 is involved in the regulation of its glycosylation and ubiquitination. Chan et al. demonstrated that PD‐L1 is phosphorylated at Y112 by JAK1, which binds to STT3A and promotes PD‐L1 glycosylation and stabilizes PD‐L1. The serine/threonine kinase AMPK phosphorylates PD‐L1 at S195 in the endoplasmic reticulum, leading to abnormal PD‐L1 glycosylation. Regarding PD‐L1 ubiquitination, the phosphorylation of PD‐L1 at S283 by AMPK disrupts the interaction between PD‐L1 and CMTM4, consequently triggering PD‐L1 ubiquitination. GSK3α mediates phosphorylation of PD‐L1 at S279 and S283, enhancing its binding to E3 ubiquitin ligase ARIH1, resulting in PD‐L1 degradation via the ubiquitin‐proteasome pathway. Consistent with the roles of AMPK and GSK3α, we found that STK25‐mediated phosphorylation of PD‐L1 at Ser283 led to PD‐L1 degradation through the ubiquitin‐proteasome pathway, and this process requires the involvement of the E3 ubiquitin ligase NEDD4.

Increasing evidence demonstrates that the serine/threonine kinase STK25 can directly bind to GOLPH3 or 14‐3‐3ζ, and regulate the phosphorylation of STAT3, YAP1, and MST1/2.^[^ ^17^, ^21^, ^22^, ^23^, ^47^ ^]^ Thus, these proteins, together with our finding of PD‐L1, might be the potential substrates of STK25, which orchestrate various cellular functions, including glycolysis, autophagy, metastasis, and tumor immune evasion.

In recent years, antitumor immunotherapy targeting the PD‐1/PD‐L1 checkpoint has demonstrated substantial clinical benefit in a series of cancers, including melanoma, NSCLC, TNBC, and CRC.^[^ ^48^, ^49^, ^50^ ^]^ Nevertheless, selecting the optimal patients for treatment remains a major challenge due to the limited response rates and potential adverse side effects. Previous studies have suggested that patients with elevated PD‐L1 levels on tumor cells may exhibit a favorable response to ICB therapy.^[^ ^51^ ^]^ The FDA has approved the application of PD‐L1 immunohistochemistry as a companied diagnostic marker for anti‐PD‐1 therapy in NSCLC patients.^[^ ^51^, ^52^ ^]^ In patients with CRC, microsatellite instability (MSI) status is a main predictor of clinical response to ICB therapy. However, in a clinical trial, the overall efficacy rate of PD‐1 blocking therapy in MSI‐H/dMMR CRC patients was less than 50%.^[^ ^3^ ^]^ Accordingly, there is a need for more accurate prognostic parameters to guide ICB treatments. In this study, we observed that STK25 deficiency rendered tumors more susceptible to ICB in STK25^−/−^ mice. CRC patients under immunosuppressive status with low STK25‐expressed tumors demonstrated heightened sensitivity to ICB therapy. Given the role of STK25 on PD‐L1 expression regulation, these findings imply that STK25 may be a useful indicator to identify patients who would benefit from ICB treatment, and a possible therapeutic target for the improvement of ICB therapy.

These findings imply that STK25 may be a useful indicator to identify patients who would benefit from ICB treatment, and a possible therapeutic target for the improvement of ICB therapy.

Although there are no clinically available inhibitors that specifically target STK25, the kinase inhibitor dasatinib, which targets SRC and BCR‐ABL, exhibited inhibitory activity against STK25 in biochemical assays.^[^ ^53^ ^]^ Studies in various tumors have demonstrated synergistic antitumor effects when combining dasatinib with ICB therapy. These benefits predominantly derived from dasatinib's modulation of tumor microenvironment components, including myeloid cells, cancer‐associated fibroblasts, and Treg cells, rather than direct STK25 pathway inhibition.^[^ ^54^, ^55^, ^56^ ^]^ Notably, a recently developed hepatocyte‐specific antisense oligonucleotide (ASO) targeting STK25 significantly reduced tumor burden in NASH‐associated hepatocellular carcinoma models.^[^ ^57^ ^]^ Further investigation is needed to determine whether the STK25‐targeting ASO has similar effects in combination with ICB therapy.

Although our study observed that systemic STK25 knockout promotes CRC tumorigenesis, our data demonstrate that targeted STK25 depletion synergizes with ICB therapy to inhibit tumor progression. The underlying mechanism likely involves STK25 depletion upregulating PD‐L1 expression levels in tumor cells, thereby remodeling TME and sensitizing previously ICB‐insensitive tumors. Indeed, our findings showed that combined sgSTK25 and ICB treatment markedly increased the infiltration of activated CD8^+^ T cells within the tumors, indicating an immunologically active TME. In line with our findings, although TRAPPC4 overexpression induces the overgrowth of tumors with a suppressive TME by upregulating PD‐L1, TRAPPC4‐overexpressed tumors achieved better efficacy with PD‐L1 blockade treatment. Thus, the application of the combination therapy in certain occasions needs further investigation.

It has been reported that a higher density of the infiltration of CD3^+^ CD8^+^ T cells is correlated with a favorable response to anti‐PD‐1 therapy,^[^ ^58^ ^]^ whereas a decrease in the intertumoral CD8^+^ T cell accumulation is linked to anti‐PD‐1 resistance in patients with MSI‐H/dMMR CRC.^[^ ^59^ ^]^ However, the reduced infiltration of cytotoxic CD8^+^ T cells in tumors with elevated Trappc4 expression may be rescued in response to anti‐PD‐1/PD‐L1 ICB therapy,^[^ ^28^ ^]^ as evidenced by the presence of pronounced CD8^+^ T‐cell infiltration, indicating enhanced antitumor immunity. Consistently, our data showed that a decrease in the abundance of CD3^+^ CD8^+^ T cells in STK25 low‐expressing tumors was associated with a superior response to anti‐PD‐1 therapy. While these discrepancies could result in suboptimal therapeutic outcomes and biased patient selection, our findings raised the possibility that specific biomarkers may serve as useful indicators for predicting ICB treatment response in patients with low CD3^+^ CD8^+^ T cell infiltration.

There are several limitations in our study. This study employed STK25 global knockout mice. The number or composition of T cells in the spleen were comparable between WT and STK25^−/−^ groups (Figure S8A, Supporting Information). Additionally, the database analysis revealed that STK25 is predominantly expressed in the non‐immune cells, compared with the immune cells (Figure S8B–E, Supporting Information). These data raise the possibility that STK25 does not directly affect systemic immune tissues. However, it has been demonstrated that STK25 is expressed and exerts functions in other cell types besides epithelial cells. STK25 in smooth muscle cells has been proved to regulate lipid accumulation and aortic oxidative stress.^[^ ^60^ ^]^ Therefore, future studies using intestinal conditional knockout mice will be valuable to obtain a comprehensive understanding of the function of STK25 in CRC initiation. Moreover, the present study identified the association between the STK25 and PD‐L1 expression in human CRC tissues. However, large cohorts of CRC patients with detailed clinical characteristics are required to further validate our findings. Furthermore, given the promising results observed with the combination of STK25 depletion and ICB treatment, additional research is needed to ascertain the potential of STK25‐targeted therapy for CRC patients undergoing immunotherapeutic regimens.

In conclusion, our study provides novel insights into the regulatory mechanism of PD‐L1 and establishes a pivotal connection between STK25 and PD‐L1‐mediated tumor immune evasion. We also demonstrate that STK25 deficiency markedly enhances the efficacy of ICB therapy. These findings highlight the potential of STK25 as a promising target for augmenting the efficacy of ICB treatment.

## 4 Experimental Section

### Patients and Tissue Samples

The patient tissues were collected from the Department of Gastrointestinal Surgery IV at Peking University Cancer Hospital & Institute, with prior approval granted by the Research Ethics Committee (approval number: 2021KT136) and in accordance with the ethical principles outlined in the Declaration of Helsinki. Prior to tissue collection, written informed consent was obtained from individual participant, ensuring that their rights were respected throughout the research process.

### Plasmids and siRNA Oligos Transfection

The plasmids containing the STK25 wild‐type and mutant (K49R/D158N) sequences were previously constructed in the laboratory.^[^ ^22^ ^]^ The NEDD4 overexpression plasmid (CH875259) was purchased from WZ Biosciences Inc. PD‐L1 WT and PD‐L1 S283A plasmids were gifts from Wenyi Wei at Harvard Medical School. Dharmacon supplied the human STK25‐specific siRNA (ON‐target plus SMARTpool, Cat #L‐004873‐00‐0005) for knockdown experiments. The siRNA is a mixture of four siRNAs provided as a single reagent. The target sequences include CUAAAGAGCACCAAGCUAU, UCUACAAGGGCAUCAUCGAUAA, ACACGCAGAUUAAGAGGAA and GCACUGGACUUGCUUAAAC. CRC cells were cultured and transfected with the corresponding plasmids or siRNAs using Lipofectamine 2000 (11668019, Invitrogen). With regard to lentiviral transfection, cells were selected with 2 µg mL^−1^ puromycin following infection with the lentivirus.

### Flow Cytometry Analysis

The apoptotic rate was evaluated by staining with Annexin V‐ FITC and propidiumiodide (PI) (Solarbio, Beijing, China) and analyzed via Flow Jo software. Apoptotic cells include both early and late apoptotic cells, as defined by Annexin V‐FITC and PI positivity. For the analysis of cell surface molecules of human and mouse T cells, cells were prepared and stained with corresponding antibodies listed in Table S5 (Supporting Information). Human T cells were stained as previously described.^[^ ^10^ ^]^ For mouse tumor tissues, single‐cell suspensions of CT26‐subcutaneous tumors and STK25^−/−^ mice CRC tumors were acquired by quick and delicate stripping, grinding, and filtering. Following Fc receptor blocking, the cells were inculcated with Zombie, CD45, CD3, CD4, CD8a, PD1, and GZMB‐specific antibodies. Stained cells were detected using Beckman CytoFLEX (Life Sciences), and the data were subsequently analyzed using Flow Jo software (version 10.0).

### CHX Chase Assay and Ubiquitination Assay

The CHX experiment was performed in accordance with the procedure previously described.^[^ ^61^ ^]^ CRC cells were transfected with siSTK25, flag‐STK25, or K49R/D158N mutation plasmids. After 36 h, cells were cultured with 100 µg mL^−1^ CHX (BN24021, Biorigin) for various time intervals as indicated. Cells were harvested, and the protein stability of PD‐L1 was measured using Western blot. For the ubiquitination assay, the HEK293T cells were transfected with indicated plasmids (Myc‐PD‐L1, Flag‐STK25, HA‐Ub‐WT, HA‐K48O‐Ub, HA‐K63O‐Ub, HA‐K27O‐Ub, HA‐K48R‐Ub, HA‐K63R‐Ub, HA‐K27R‐Ub) and treated with MG132 (HY13259, MCE). Then the cells were harvested and the total protein was extracted. Immunoprecipitation was conducted using a PD‐L1‐specific antibody. The ubiquitinated PD‐L1 proteins were assessed through Western blot with the anti‐ubiquitin antibody.

### Animal Experiments

Homozygous STK25 whole body knockout (STK25^−/−^) mice on a C57BL/6 background were constructed via CRISPR/Cas9 methods by the Research Center of the Southern Model Organism. Female BALB/c mice were purchased from the Hua Fukang Bioscience Company. The mice were housed under SPF conditions with a 12 h light/12 h dark cycle and provided a standard diet. Mice aged 6 to 8 weeks, weighing 18–20 g, were used for all experiments. All experiments involving mice were carried out in accordance with the Ethics Committee of the Peking University Cancer Hospital & Institute (2021KT134).

Azoxymethane (AOM)/dextran sulfate sodium (DSS)‐induced CRC and PD‐1mAb treatment: STK25^−/−^ and wild‐type mice (6‐week‐old, sex‐matched) were administered intraperitoneally with a single dose of 10 mg kg^−1^ AOM (A5486, Sigma–Aldrich). One week following the AOM treatment, all mice (n = 10 per group)were subjected to three cycles of DSS (0216011080, MP Biomedicals) treatment (2% in water for 5 consecutive days), with a 2‐week rest period between each cycle. The mice were sacrificed 120 days following the initial AOM injection. The colons were harvested, measured for length, prepared as Swiss rolls, and stored in 4% paraformaldehyde (PFA) or liquid nitrogen. As for the PD‐1 mAb intervention experiment, STK25^−/−^ and wild‐type mice were administered IgG or PD‐1 mAb isotype therapy as previously mentioned 120 days after the AOM injection (n = 3 per group). For blockade of PD‐1/PD‐L1 signaling, PD‐1 mAb (100 µg) (BE0146, Bio X Cell) or IgG isotype was injected intraperitoneally on days 7, 11, 14, 18, 21, and 25.

Subcutaneous tumors and PD‐1 mAb treatment: SgSTK25 or control CT26 cells (2×10^5^ cells) were subcutaneously inoculated into the flanks of BALB/c mice. Once the tumor reached an average volume of 100 mm^3^, all mice were randomly divided into four groups (n = 5 per group): (a) CTRL+IgG, (b) sgSTK25+IgG, (c) CTRL+anti‐PD‐1 antibody, and (d) sgSTK25+anti‐PD‐1 antibody. The tumor volume was recorded every 4 days using the formula: 1/2 × length × width^2^. The mice were sacrificed via CO_2_ asphyxiation once the tumor burden reached 1500 mm^3^. Tumor tissues were collected for photography, weighing, and embedding. Additionally, the tumors were dissociated and processed into single‐cell suspensions for subsequent FACS analysis.

CD8^+^ T cell depletion assay: Mice were intraperitoneally injected with 100 µg αCD8α (A210225, Selleck) or IgG isotype control in combination with PD‐1 mAb therapy, administered every three days for two weeks. The effectiveness of CD8^+^ T cell depletion was validated by flow cytometry analysis of tumor tissues and spleen.

### Mouse‐Derived Organoid

Mouse intestinal cancer organoids were processed as described before.^[^ ^62^ ^]^ In brief, mouse colon tumor tissues were chopped into small pieces, rinsed with PBS buffer, and then incubated in an EDTA digestion solution. The digest was centrifuged, resuspended in Matrigel (356 255, Corning), and 20 µL of the mixture was added to each well of a preheated 48‐well plate. Once the mixture solidified, 300 µL of mouse intestinal organoid medium (MA‐0807T001LP, Mogengel) was added to each well. The medium was replenished every 2–3 days. The size of the organoids was assessed on day 9 and analyzed using Image J.

### Single‐Cell RNA Sequencing (scRNA‐seq)

Fresh CRC tissues were obtained from the intestines of wild‐type and STK25^−/−^ mice. The CRC tissues derived from the same group of mice were pooled together for subsequent analysis. The tissues were promptly minced with scissors and subjected to enzymatic digestion with collagenase‐type IV‐containing digestion solution at 37 °C for 30 min. Then, the cell suspension was passed through a 45 µm mesh to generate single‐cell suspension. The single cells from both samples were loaded onto a microwell chip for reverse transcription and construction of cDNA libraries. An Illumina NovaSeq 6000 sequencer was used to accomplish the sequencing, which had a minimum sequencing depth of 100 000 reads per cell and 150 bp paired‐end reads.

### Statistical Analysis

GraphPad Prism 8 was used for the statistical analysis. For comparisons between two groups, an unpaired Student's t test was employed to ascertain statistical significance. Two‐way ANOVA was used for multiple comparisons. All experiments were performed at least three times. Data are presented as mean ± SD, and a *p*‐value of less than 0.05 was considered statistically significant.

## Conflict of Interest

The authors declare no conflict of interest.

## Author Contributions

X.Q. and P.X. contributed equally to this work and are co‐first authors. X.Q., P.X., H.H., J.C., L.S., Y.H., and X.Y. performed conceptualization, investigation, formal analysis, and methodology. K.W., J.C., P.G., T.S., H.Y., T.L., Y.R., B.C., W.Z., J.D., and Z.W. performed software and visualization. X.Q., P.X., J.Z., and B.J. wrote the original draft, reviewed, and edited the final manuscript. J.C., H.Y., W.Z., J.Z., X.S., and B.J. acquired funding acquisition. All authors approved the final version of the manuscript.

## Supporting information



Supporting Information

Supporting Information

Supplemental Table 1

Supplemental Table 2

Supplemental Table 3

Supplemental Table 4

Supplemental Table 5

Supplemental Table 6

Supporting Information

## Data Availability

ScRNA‐seq raw sequencing files can be found in the GEO database (GSE277814). Any additional data can be requested from the corresponding author.
