# Supplementary material for: STK25 Loss Augments Anti‐PD‐1 Therapy Efficacy by Regulating PD‐L1 Stability in Colorectal Cancer
Source: Adv Sci (Weinh). 2025 Jul 29;12(39):e03891. doi: 10.1002/advs.202503891 (PMC12533155; doi:10.1002/advs.202503891)
Supplement: Supplementary file 1 — Supporting Information [file ADVS-12-e03891-s004.docx]

STK25 Loss Augments Anti-PD-1 Therapy Efficacy by Regulating PD-L1 Stability in Colorectal Cancer

*Xiaowen Qiao^1^*^†^*, Pu Xing^1,2^*^†^*, Hao Hao^1^, Jiangbo Chen^1^, Lin Song^1^,Yifan Hou^1^, Xinying Yang^1^, Kai Weng^1^, Jie Chen^3^, Pin Gao^1^, Tongkun Song^1^, Hong Yang^1,4^, Tianqi Liu^1,5^, Yumeng Ran^1^,*

*Bo Chen^1^, Wei Zhao^6^, Jiabo Di^1^, Zaozao Wang^1^, Jun Zhang^7*^, Xiangqian Su^1,8*^, Beihai Jiang^1*^*

*Corresponding authors.

**Supplementary methods**

**Cell culture:** The mouse CRC cell lines (MC38, CT26) and human CRC cell lines (RKO, LoVo) were obtained from the ATCC. Cells were cultured at 37 °C in a 5% CO_2_ environment in RPMI-1640 or DMEM medium (SH30809, SH30022 HyClone) with 10% FBS, 100 units/mL of penicillin, and 100μg/mL of streptomycin.

**Generation of STK25 knockout cells:** We utilized CRISPR–Cas9 lentiviruses (Jikai Gene) to knockout STK25. To achieve human STK25 knockout, the following sgRNA sequences were used: sgSTK25#1 5′-TCATCGACCGCTATAAGCGC-3′, and sgSTK25#2 5′-GGGGATCACAGCCATCGAGC-3′. To achieve mouse STK25 knockout, the following sgRNA sequences were used: sgSTK25#1 5′-TTCACCAAGCTTGACCGCAT-3′, and sgSTK25#2 5′-CCGAAGTAGCGGGTGATATA-3′. Lentiviruses containing the sgRNA sequences were transfected into CRC cells for 48 hours. The culture media was then supplemented with 4 μg /mL of puromycin. After one week, the remaining viable cells were selected individually into 96-well plates. These subclones were amplified, and the knockout effect was verified by western blot.

**Co-culture assay:** Whole blood samples were provided by healthy donors. Peripheral blood mononuclear cells (PBMCs) were isolated from whole blood samples provided by healthy donors using Ficoll (07801, Stemcell) according to the manufacturer’s instructions. RPMI 1640 media with 10% FBS were used to maintain the cells. The human T cell activator anti-CD3/CD28 (10971, Stemcell) and IL-2 (78036, Stemcell) were supplemented to the culture medium (5 μg/mL and 10 ng/mL, respectively). The mouse T cells were obtained from the spleen of BALB/c mice. In brief, mouse splenocytes were incubated in medium containing 2 μg/mL anti-CD3 antibody (100340, BioLegend), 1mg/mL anti-CD28 antibody (102116, BioLegend), and 100ng/mL recombinant mouse IL-2 (575404, BioLegend). At 7 days after stimulation, T cells were used in a tumor cell killing assay. CRC tumor cells (LoVo, RKO, CT26 cell) were co-cultured with T cells in 6 wells plates for 48 hours at effector-to-target ratio of 1:5. Then, T cells were washed out, and the adherent CRC cells were either harvested for an Annexin-V/PI apoptosis assay or stained with 1% crystal violet.

**Enzyme-linked immunosorbent assay (ELISA):** The supernatant was harvested from the tumor cell-T cell co-culture system. The supernatants were diluted 10- to 50-fold for ELISA analysis according to the manufacturer's instructions. IL-2 (1110202) and IFN-γ kits (1110002) were purchased from Dakewe Bioengineering Co., Ltd.

**Quantitative real-time PCR (qRT-PCR):** Total RNA was extracted from CRC tissues or cells using the TRIzol reagent (15596018, Thermo Fisher Scientific). cDNA was generated by a reverse transcription kit (A5004, Promega). Quantitative RT-PCR was performed on an ABI 7500 PCR System using SYBR Green PCR Master Mix (QPK-201, Toyobo). The primers employed in this study are listed in Supplementary Table S6. GAPDH was used as the endogenous control for normalization.

**Western blot:** The cells were lysed by cell lysis buffer containing 50mM Tris-HCL (pH8.0), 150mM NaCL, 1mM EDTA, 1% Triton X-100, 0.5% NP-40, proteinase inhibitor and phosphatase inhibitor. The concentrations of total protein were detected using a Bradford assay kit (5000205, Bio-Rad). Equal amounts of proteins from each group were separated according to standard protocol. Subsequently, protein bands were incubated with the corresponding antibodies listed in Supplementary Table S5. The visualization of proteins was conducted via chemiluminescence using an ECL Western blot detection kit (WBKLS0100, Millipore) and AI600 imaging system (GE Healthcare).

**Analysis of scRNA-seq Data:** The 10x Genomics sequencing data were aligned and quantified based on the mouse reference genome (mm10) using the Cell Ranger software package (version 3.1). Subsequently, the Seurat R package was used for processing the scRNA-seq data. Cells with fewer than 200 detected genes, mitochondrial gene contributions exceeding 25%, or mitochondrial RNA content greater than 20% were excluded from further analysis. After dimensionality reduction using principal component analysis (PCA), clusters in the low-dimensional space were visualized using uniform manifold approximation and projection (UMAP). The cells were classified into seven clusters based on specific markers, including epithelial cells (Epcam, Cdh1, Krt8, Krt18, Krt19, Atoh1, Muc2, Reg4, Guca2a), fibroblasts (Col1a2, Col1a1, Dcn, Sparc), endothelial cells (Pecam1, Cdh5, Ramp2, Eng), neutrophils (Csf3r, Cxcr2, Ly6g, Mmp9, S100a8), macrophages (Cd74, H2-DMa, H2-Eb1, H2-DMb1, H2-Ab1, H2-Aa), T cells (Cd3d, Cd3e, Trbc2, Cd3g, Icos), and B cells (Cd79a, Cd79b).

**Co-immunoprecipitation (Co-IP) assays:** The total protein from the transfected cells was incubated with Protein A/G beads for 2 hours in order to perform Co-IP analysis with the indicated antibodies. Subsequently, 2µg of antibody was used for immunoprecipitation, which was conducted overnight at 4 °C. Following carefully cleaning the beads in ice-cold lysis solution, a Western blot analysis was performed. The coimmunoprecipitations were detected by immunoblotting with the specified antibodies.

**Integrated Identification of NEDD4 as an STK25-PD-L1 Scaffold:** Firstly, experimentally validated STK25-interacting proteins were extracted from the “Interactions” section of NCBI’s Gene database (https://www.ncbi.nlm.nih.gov/gene/10494). Secondly, the top 20 PD-L1-targeting E3 ubiquitin ligases were retrieved from UbiBrowser (http://ubibrowser.bio-it.cn/ubibrowser/strict/networkview/networkview/name/Q9NZQ7/jobId/ubibrowse-I2025-06-26-43498-1750941686), a database predicting human E3-substrate interactions. Lastly, overlapping candidates from both datasets were considered potential regulatory E3 ligases in this pathway.

**Immunohistochemical (IHC) staining:** CRC tissue samples embedded in paraffin from patients or mice were sectioned into 4 μm slices. Following deparaffinization, endogenous peroxidase was blocked, and antigen retrieval was conducted. All sections were incubated with corresponding primary antibodies at 4°C overnight. The detailed information of antibodies was listed in Supplementary Table S5. Subsequently, the sections were incubated with secondary antibodies for 1 hour, subjected to DAB staining, and hematoxylin counterstaining. The intensity of IHC staining was rated on a scale from 1 to 4, corresponding to negative, weak, moderate, and strong staining, respectively. The extent of staining was graded on a scale of 0-25%, to 25-50%, to 50-75%, and to 75-100% based on the proportion of positive cells. The stain intensity and stain extent measurements were multiplied to determine the final IHC score. The median expression value of the final IHC score was used to categorize the CRC samples into groups with low and high expression. The results analyzed by the two independent pathologists showed a high level of consistency. Any disagreements were re-examined to reach at a final determination.
