# Supplementary material for: STK25 Loss Augments Anti‐PD‐1 Therapy Efficacy by Regulating PD‐L1 Stability in Colorectal Cancer
Source: Adv Sci (Weinh). 2025 Jul 29;12(39):e03891. doi: 10.1002/advs.202503891 (PMC12533155; doi:10.1002/advs.202503891)
Supplement: Supplementary file 2 — Supporting Information [file ADVS-12-e03891-s003.pdf]

ADVANCED SCIENCE
Open Access

Supporting Information

for *Adv. Sci.*, DOI 10.1002/advs.202503891

STK25 Loss Augments Anti-PD-1 Therapy Efficacy by Regulating PD-L1 Stability in Colorectal Cancer

Xiaowen Qiao, Pu Xing, Hao Hao, Jiangbo Chen, Lin Song, Yifan Hou, Xinying Yang, Kai Weng, Jie Chen, Pin Gao, Tongkun Song, Hong Yang, Tianqi Liu, Yumeng Ran, Bo Chen, Wei Zhao, Jiabo Di, Zaozao Wang, Jun Zhang, Xiangqian Su* and Beihai Jiang**

Supplementary Figure 1. Related to Figure 1.

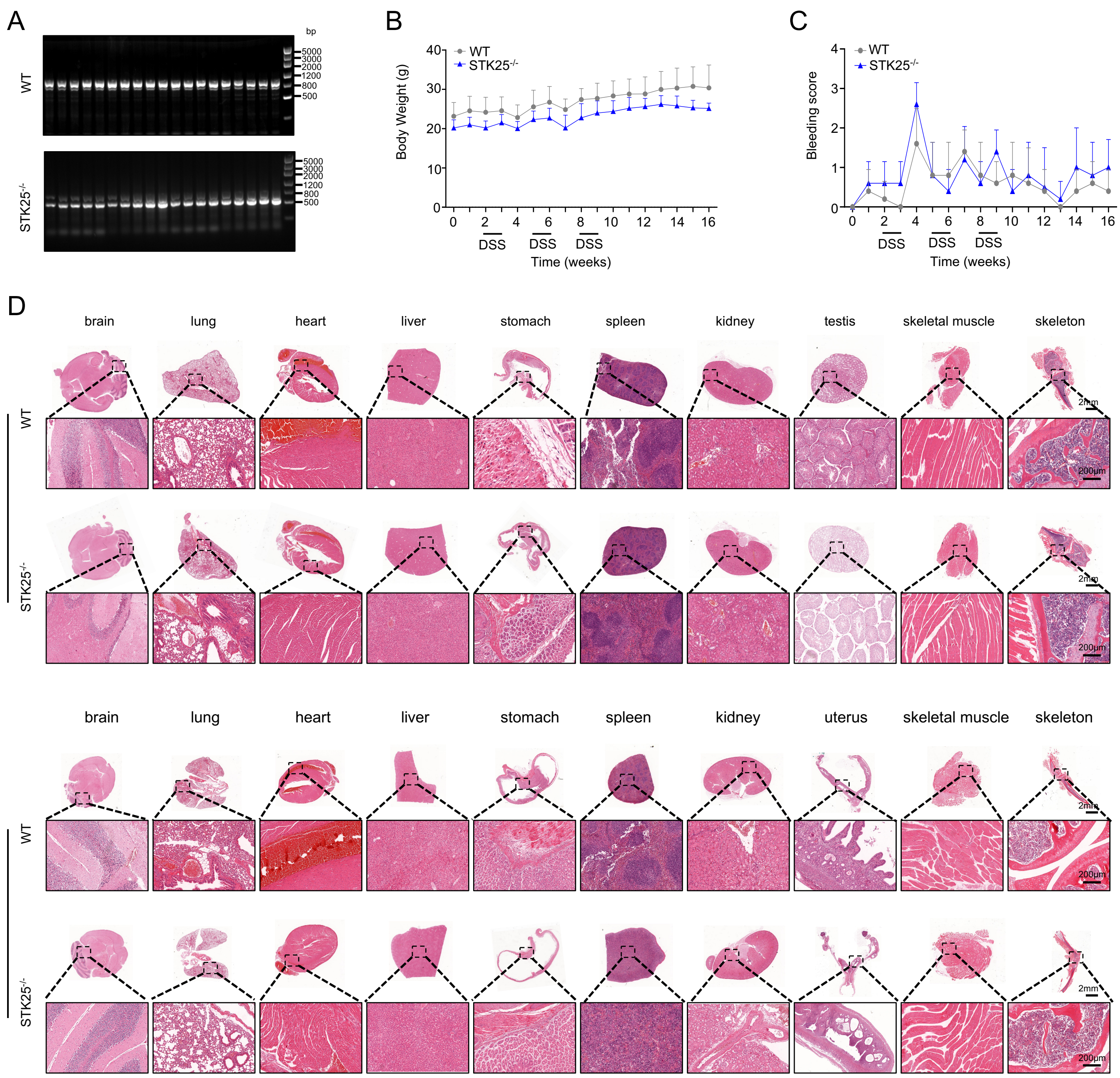

Supplementary Figure 1. STK25 global knockout (STK25$^{-/-}$) in mice has no distinct effect on the body weight and fecal occult blood scores. (A) Genotype identification using PCR analysis of DNA from normal WT and global knockout mice. **(B, C)** Effects of STK25 deficiency on AOM/DSS-induced body weights (B) and bleeding score changes (C). **(D)** Pathological analysis of several organs of the WT and STK25$^{-/-}$ mice by H&E staining. Scale bars, 2 mm and 100 μm.

Supplementary Figure 2. Related to Figure 2.

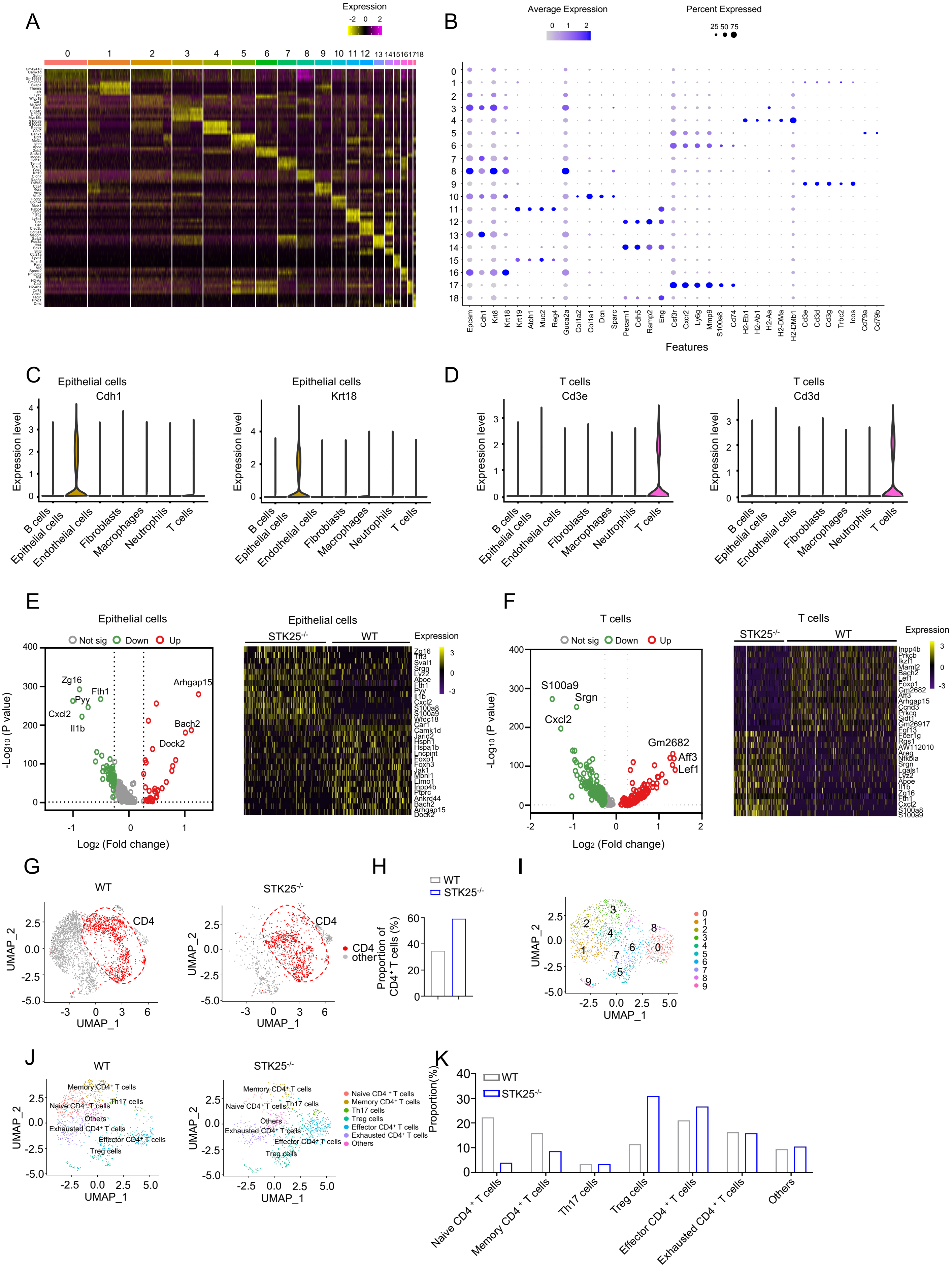

Supplementary Figure 2. The differential expression genes (DEGs) between WT and STK25$^{-/-}$ mice according to the scRNA-seq analysis. (A) Heatmap of the most differentially expressed marker genes in each cell clusters of our scRNA-seq data. **(B)** The expression of the marker genes used for the cell type annotation is indicated on the DotPlot. **(C, D)** Violin plots depict expression of the marker genes used to annotate epithelial cells (C) and T cells (D). **(E, F)** Volcano plot (left) and heatmap (right) showing the DEGs in epithelial cells (E) or T cells (F) between WT and STK25$^{-/-}$ mice**.(G, H)** Density plots of CD4^{+} T cells in T cells from WT and STK25$^{-/-}$ mice after AOM/DSS treatment (G). (H) Corresponding bar graphs of (G) show quantification of CD4^{+} T cell percentage (H). **(I)** UMAP plot of ten CD4^{+} T cell subsets in CRC of mice, with each cell color coded for cell clusters. **(J)** UMAP plot of CD4^{+} T cell subsets in WT and STK25$^{-/-}$ mice treated with AOM/DSS. **(K)** The proportions of each cell type (J) in WT and STK25$^{-/-}$ mice treated with AOM/DSS.

Supplementary Figure 3. Related to Figure 3.

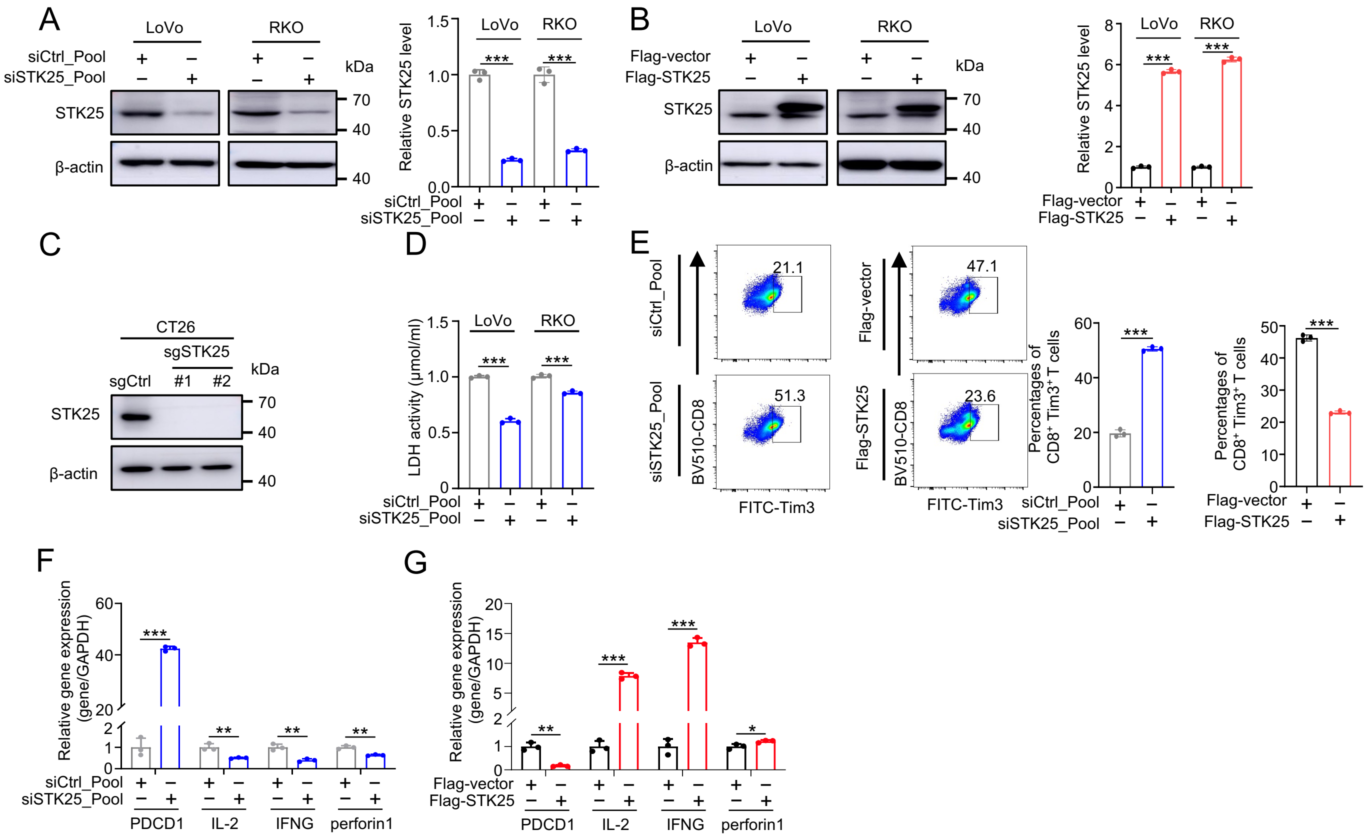

Supplementary Figure 3. STK25 deficiency in CRC cells significantly inhibits the cytotoxic effects of T cells. (A-C) The knockdown (A), overexpression (B), and knockout (C) efficiency were validated using Western blot analysis. **(D)** An LDH-releasing assay was performed to assess the activity of CRC cells that were co-cultured with PBMCs. **(E)** The plots and graphs showed percentages of $CD8^+$ $Tim3^+$ T cells of PBMCs co-cultured with RKO cells transfected with siSTK25 or flag-STK25. **(F, G)** qRT-PCR showed the expression of PD-1 and cytokines, including IL-2, IFN-γ, and perforin-1, in human PBMCs that were co-cultured with siSTK25 (F) or flag-STK25 (G)-treated CRC cells. The above experiments were repeated three times independently. The data are presented as the mean ± SD, and P values were calculated using a two-way ANOVA analysis or unpaired two-sided Student's t-test. *p<0.05, **p<0.01, ***p<0.001.

Supplementary Figure 4. Related to Figure 4.

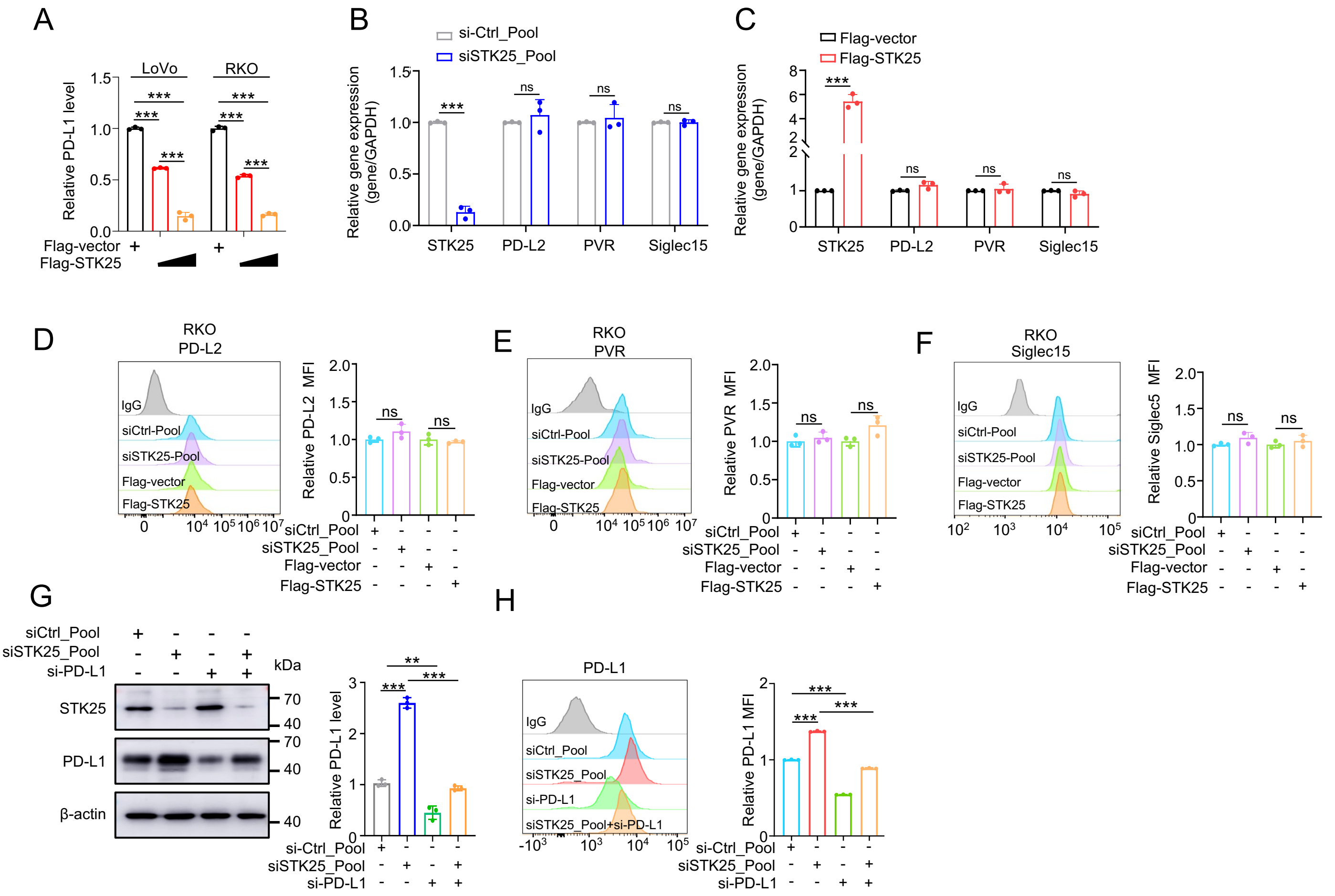

Supplementary Figure 4. STK25 can negatively regulate PD-L1 expression. (A) Quantification of relative PD-L1 protein levels normalized to β-actin in Figure 4C. **(B, C)** qRT-PCR showed the expression of STK25, PD-L2, PVR and Siglec15 in CRC cells transfected with siSTK25 (B) or flag-STK25 (C). **(D-F)** Flow cytometry analysis showed PD-L2 (D), PVR(E) and Siglec15 (F) levels on CRC cell transfected with siSTK25 or flag-STK25. Bar graphs show the mean fluorescence intensity (MFI). **(G, H)** Verification of siRNA PD-L1 knockdown efficiency in siSTK25 CRC cells through Western blot (G) and flow cytometric analysis (H). The above experiments were repeated three times independently. The data are presented as the mean ± SD, and P values were calculated using an unpaired two-sided Student's t-test **p<0.01, ***p<0.001; ns, not significant.

Supplementary Figure 5. Related to Figure 5.

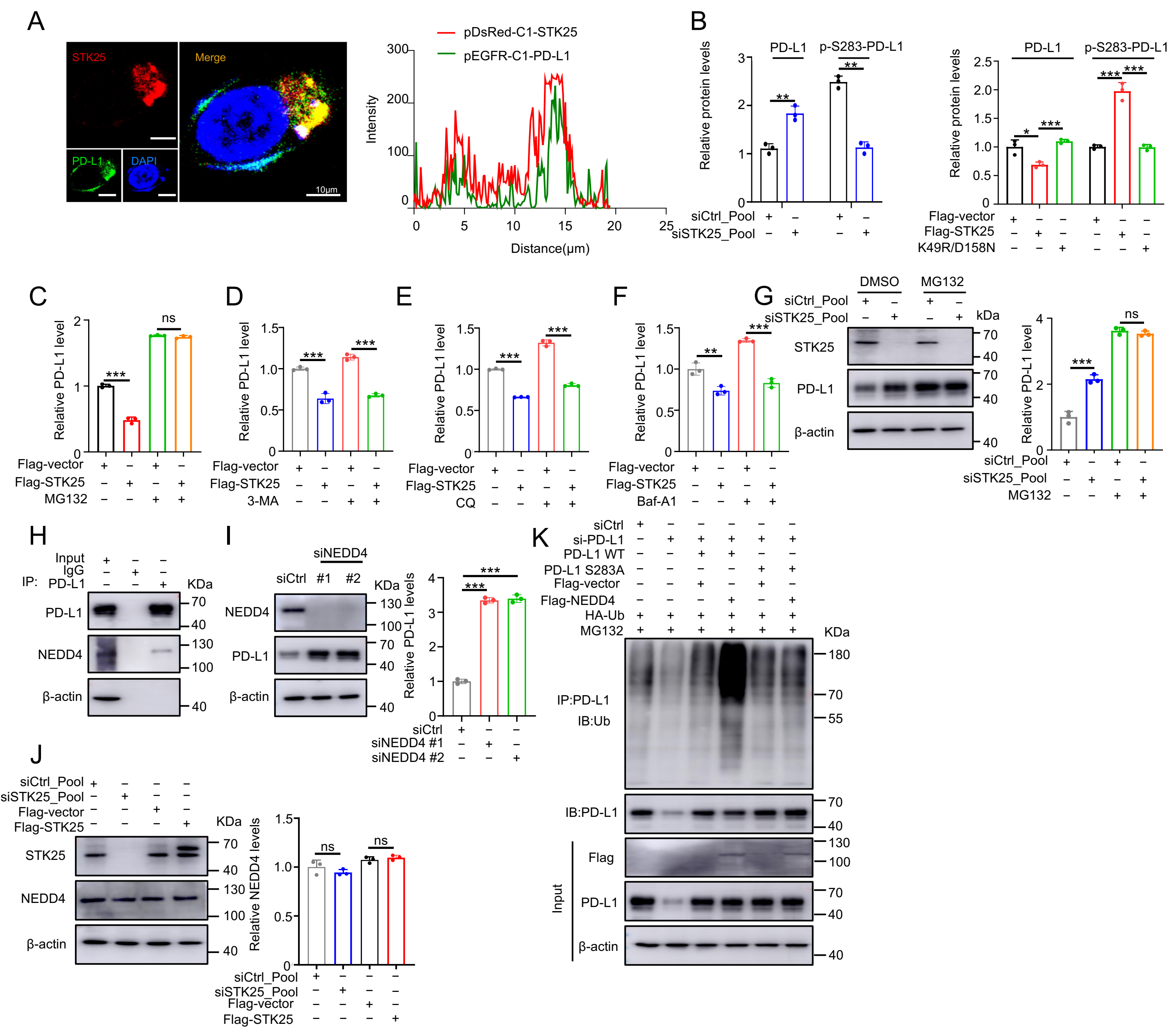

Supplementary Figure 5. STK25 regulates PD-L1 levels in a NEDD4-dependent manner. (A) Cells co-transfected with pDsRed-C1-STK25 and pEGFP-C1-PD-L1 were observed under a confocal microscope. Colocalization was shown by merge (yellow). Fluorescence intensity graph (bottom) shows the level of co-localization. Scale bar, 10 μm. **(B)** Quantification of relative protein levels of PD-L1 and p-S283-PD-L1 in Figure 5C. **(C-F)** Quantification of relative protein levels of PD-L1 in Figure 5I-L. **(G)** Western blot analysis detected the PD-L1 expression on CRC cells transfected with siSTK25 after treatment with MG132. **(H)** Interaction of endogenous PD-L1 with NEDD4 in RKO cells. Co-IP was performed using an anti-PD-L1 antibody. **(I)** Western blot analysis of NEDD4 and PD-L1 expression in the NEDD4-knockdown cells. **(J)** The protein levels of NEDD4 in RKO cells transfected with indicated siRNA and plasmids. **(K)** RKO cells were transfected with the indicated plasmids. Ubiquitinated PD-L1 was immunoprecipitated and subjected to western blot analysis with the ubiquitin antibody. Prior to the ubiquitination analysis, MG132 was administered to the cells. The above experiments were repeated three times independently. The data are presented as the mean ± SD, and P values were calculated using an unpaired two-sided Student's t-test. **p<0.01, **p<0.05, ***p<0.001; ns, not significant.

Supplementary Figure 6.Related to Figure 6.

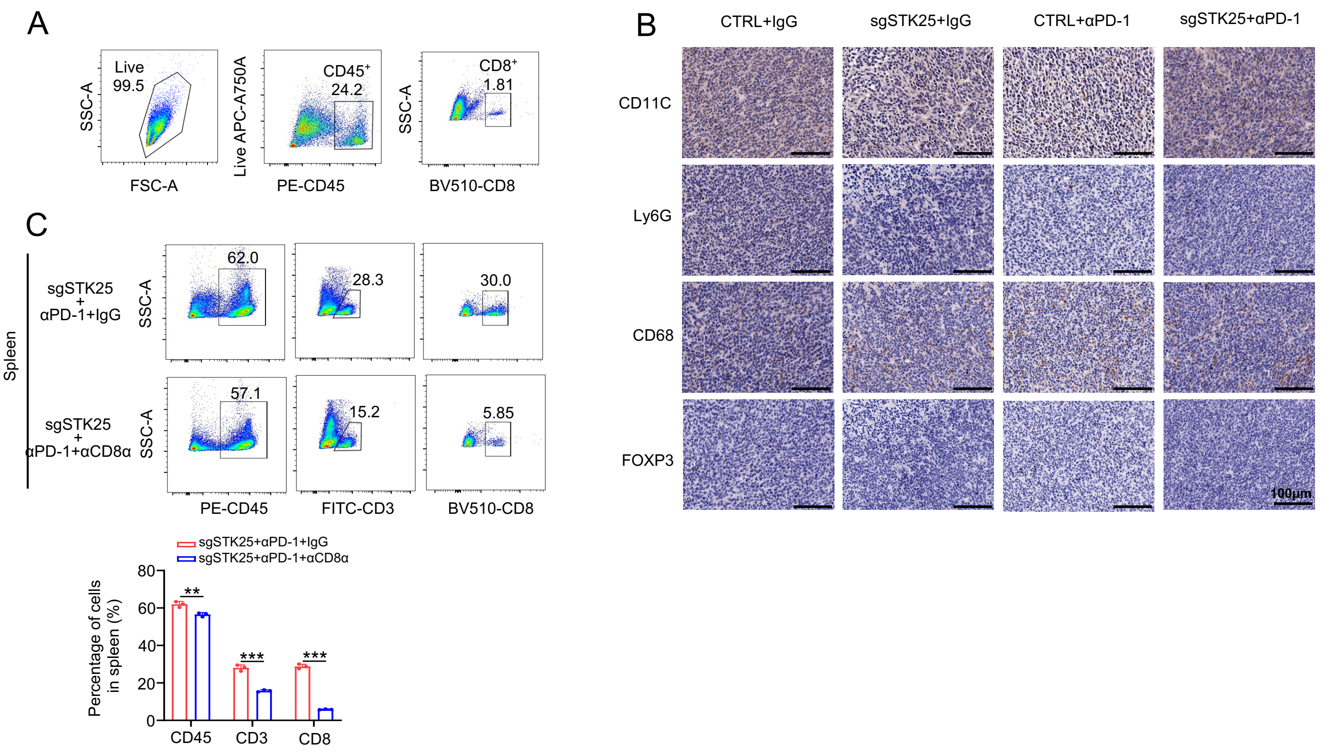

Supplementary Figure 6. Analysis of immune cell infiltration in CT26 xenografts and T cells in mice spleen. (A) Sequential gating strategy of lymphocytes from tumor tissue for flow cytometry analysis. **(B)** Representative images of IHC staining with anti-CD11C, anti-Ly6G anti-CD68 and anti-FOXP3 in sgSTK25 or Ctrl CT26 xenografts. Scale bars, 100μm. **(C)** The proportions of infiltrating $CD3^+$ T cells, $CD8^+$ T cells in $CD45^+$ cells in total splenic immune cells (n=3 per group) were detected using flow cytometry. and quantified. The experiments were repeated three times independently. The data are presented as the mean ± SD, and P values were calculated using two-sided unpaired t test. **p<0.01, ***p<0.001.

Supplementary Figure 7. Related to Figure 7.

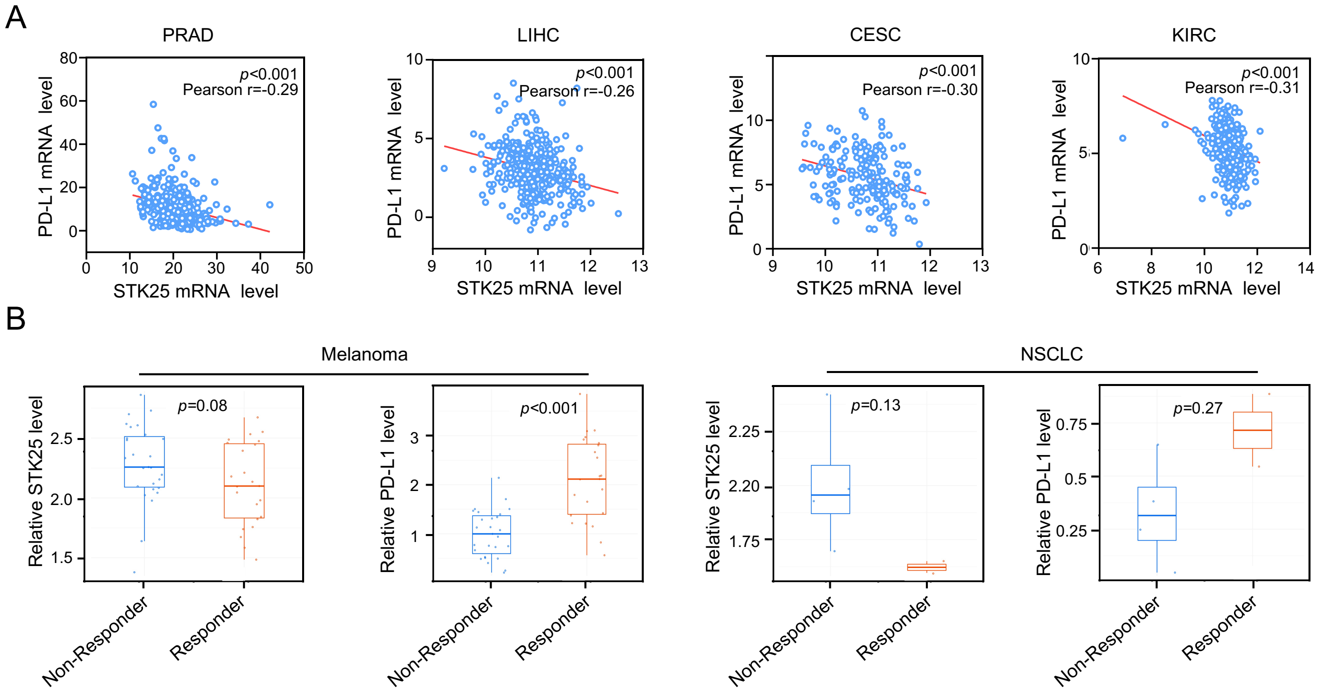

Supplementary Figure 7. The expression of STK25 is negatively associated with that of PD-L1 in CRC patient samples. (A) Correlations between STK25 and PD-L1 in various tumors were calculated using the cBioPortal tool. **(B)** The TIGER database was employed to determine the correlation of the STK25 and PD-L1 expression with the response to ICB therapy in melanoma and NSCLC, respectively.

Supplementary Figure 8

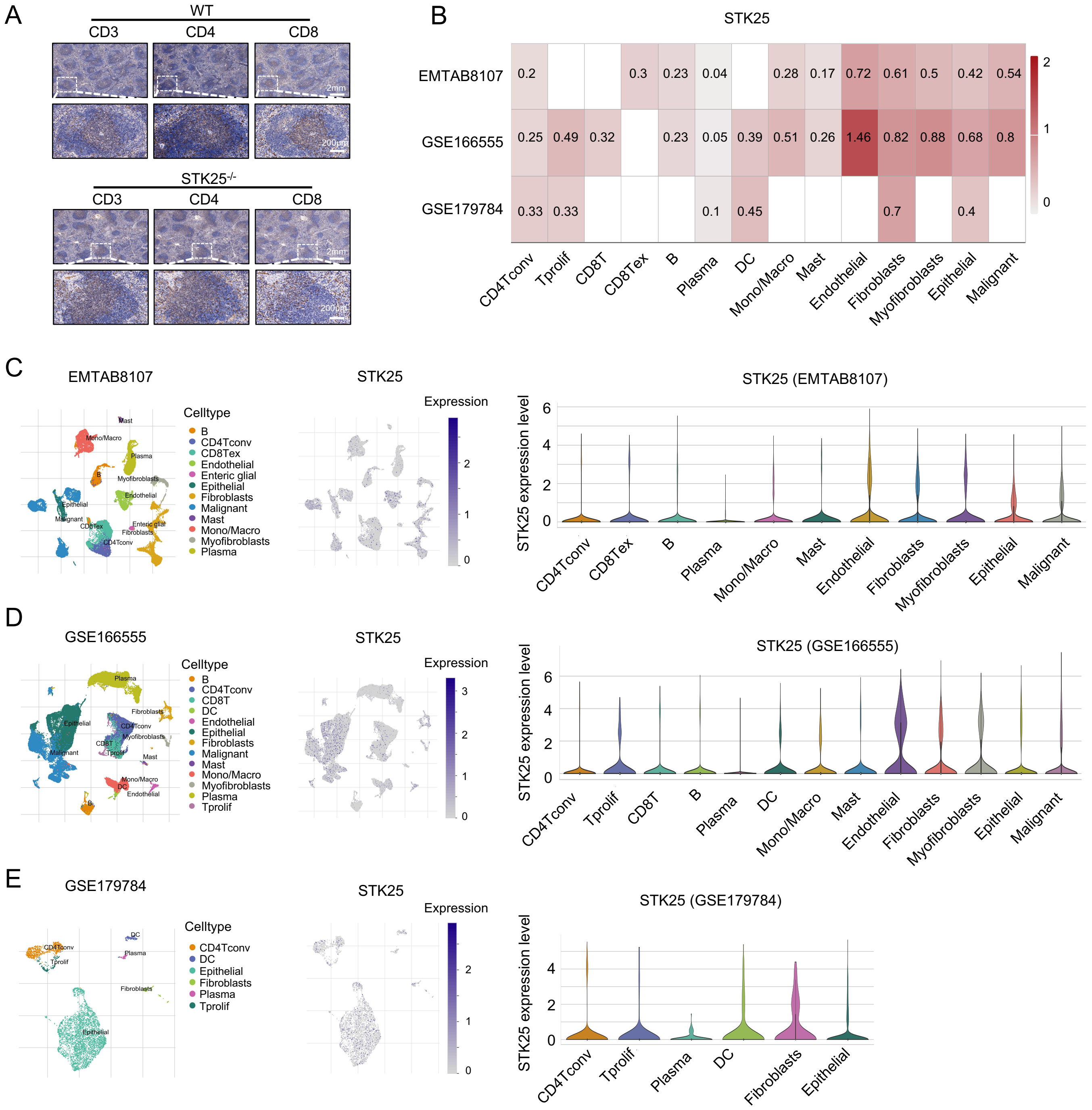

Supplementary Figure 8. The expression levels of STK25 in different cell types. (A) Immunohistochemistry staining of WT and $STK25^{-/-}$ mice spleen sections using anti-CD3, CD4, and CD8 antibodies. STK25 depletion has no significant effect on the numbers of T cells in mice spleen. Scale bars, 2 mm and 100 μm. **(B)** The expression levels of STK25 in CRC single-cell datasets from the TISCH database. **(C-E)** UMAP plot and violin plot of STK25 expression across several cell clusters in the CRC single-cell datasets, including EMTAB8107 (C), GSE166555 (D), GSE179784 (E).
