# Supplementary material for: STK25 Loss Augments Anti‐PD‐1 Therapy Efficacy by Regulating PD‐L1 Stability in Colorectal Cancer
Source: Adv Sci (Weinh). 2025 Jul 29;12(39):e03891. doi: 10.1002/advs.202503891 (PMC12533155; doi:10.1002/advs.202503891)
Supplement: Supplementary file 3 — Supplemental Table 1 [file ADVS-12-e03891-s008.docx]

STK25 Loss Augments Anti-PD-1 Therapy Efficacy by Regulating PD-L1 Stability in Colorectal Cancer

*Xiaowen Qiao^1^*^†^*, Pu Xing^1,2^*^†^*, Hao Hao^1^, Jiangbo Chen^1^, Lin Song^1^,Yifan Hou^1^, Xinying Yang^1^, Kai Weng^1^, Jie Chen^3^, Pin Gao^1^, Tongkun Song^1^, Hong Yang^1,4^, Tianqi Liu^1,5^, Yumeng Ran^1^,*

*Bo Chen^1^, Wei Zhao^6^, Jiabo Di^1^, Zaozao Wang^1^, Jun Zhang^7*^, Xiangqian Su^1,8*^, Beihai Jiang^1*^*

*Corresponding authors.

### Supplementary Table S1. Screening of E3 ligases mediating PD-L1 ubiquitination

| STK25-interacting proteins  (NCBI database) | | | | PD-L1-associated E3 ligases  (UbiBrowser database) | Overlapping E3 ligase |
| --- | --- | --- | --- | --- | --- |
| GOLGA2 | CLCN7 | IHO1 | **NEDD4** | MARCH9 | **NEDD4** |
| PDCD10 | CLEC16A | IKBKG | OGT | SYVN1 |  |
| UBQLN4 | CPVL | KCNA6 | ORF1ab | SKP2 |  |
| YWHAZ | CTTNBP2 | KIF20A | PBX2 | CBL |  |
| ACACA | CTTNBP2NL | KIFBP | PDCD10 | DTX1 |  |
| ACACB | Other Gene | LATS2 | PGCKA1 | SOCS7 |  |
| ACAD11 | DYNLL1 | LIN7C | PHKA2 | CBLC |  |
| AKAP9 | Dynll1 | LRRK2 | PLEKHA4 | MARCH5 |  |
| ANAPC16 | EIF4A3 | MAD2L1 | POLR3K | MARCH2 |  |
| ANGPT1 | EVA1C | MANF | PPP2CA | MARCH6 |  |
| APP | FGFR1OP2 | MAP4K3 | PPP2CB | MARCH1 |  |
| ARL13B | GNG2 | MAPK9 | PPP2R1A | MARCH11 |  |
| ARV1 | GOLGA2 | MFHAS1 | PPP2R1B | BTRC |  |
| ATP2B1 | GOLGA6L9 | MILR1 | PPP4C | MARCH4 |  |
| C9orf72 | GORASP1 | MOB4 | PRNP | MARCH8 |  |
| CAB39 | HBS1L | MOS | PROSER2 | MARCH3 |  |
| CCNDBP1 | HDDC3 | MTDH | RICTOR | MARCH7 |  |
| CDKAL1 | HSCB | MTMR6 | RIPOR1 | **NEDD4** |  |
| CEP70 | HSP90AA1 | MTPAP | RPL12 | PAFAH1B1 |  |
| CHMP4C | HSP90AB1 | TAX1BP1 | RYBP | FBXW11 |  |
| SERBP1 | STRN | TBC1D9 | USP36 |  |  |
| SIKE1 | STRN3 | TCP1 | YWHAB |  |  |
| SLMAP | STRN4 | TIMP2 | ZDHHC17 |  |  |
| STK24 | SUGT1 | TOLLIP | ZNF598 |  |  |
| STK26 | SYT6 | TRIM27 | STRIP2 |  |  |
| STK3 | TAOK1 | TRIM45 | TAOK3 |  |  |
| STRIP1 | TAOK2 | UBE2D3 |  |  |  |
