# Supplementary material for: STK25 Loss Augments Anti‐PD‐1 Therapy Efficacy by Regulating PD‐L1 Stability in Colorectal Cancer
Source: Adv Sci (Weinh). 2025 Jul 29;12(39):e03891. doi: 10.1002/advs.202503891 (PMC12533155; doi:10.1002/advs.202503891)
Supplement: Supplementary file 4 — Supplemental Table 2 [file ADVS-12-e03891-s007.docx]

STK25 Loss Augments Anti-PD-1 Therapy Efficacy by Regulating PD-L1 Stability in Colorectal Cancer

*Xiaowen Qiao^1^*^†^*, Pu Xing^1,2^*^†^*, Hao Hao^1^, Jiangbo Chen^1^, Lin Song^1^,Yifan Hou^1^, Xinying Yang^1^, Kai Weng^1^, Jie Chen^3^, Pin Gao^1^, Tongkun Song^1^, Hong Yang^1,4^, Tianqi Liu^1,5^, Yumeng Ran^1^,*

*Bo Chen^1^, Wei Zhao^6^, Jiabo Di^1^, Zaozao Wang^1^, Jun Zhang^7*^, Xiangqian Su^1,8*^, Beihai Jiang^1*^*

*Corresponding authors.

**Supplementary Table S2:** Clinical information for 4 CRC samples, related to Figure 7A.

| Sample ID | Gender | Age | Tumor location | T stage | N stage | M stage | Pathologic stage | Differentiation grade | Preoperative treatment |
| --- | --- | --- | --- | --- | --- | --- | --- | --- | --- |
| 1 | M | 73 | Hepatic Flexure | T4a | N0 | M0 | IIA | Low | NO |
| 2 | M | 51 | Hepatic Flexure | T4a | N1 | M1a | IVA | Middle | NO |
| 3 | F | 33 | Transverse Colon | T4a | N0 | M0 | IIA | Middle | NO |
| 4 | M | 77 | Transverse Colon | T4a | N1 | M0 | IIIB | Middle | NO |
