# Supplementary material for: STK25 Loss Augments Anti‐PD‐1 Therapy Efficacy by Regulating PD‐L1 Stability in Colorectal Cancer
Source: Adv Sci (Weinh). 2025 Jul 29;12(39):e03891. doi: 10.1002/advs.202503891 (PMC12533155; doi:10.1002/advs.202503891)
Supplement: Supplementary file 5 — Supplemental Table 3 [file ADVS-12-e03891-s002.docx]

STK25 Loss Augments Anti-PD-1 Therapy Efficacy by Regulating PD-L1 Stability in Colorectal Cancer

*Xiaowen Qiao^1^*^†^*, Pu Xing^1,2^*^†^*, Hao Hao^1^, Jiangbo Chen^1^, Lin Song^1^,Yifan Hou^1^, Xinying Yang^1^, Kai Weng^1^, Jie Chen^3^, Pin Gao^1^, Tongkun Song^1^, Hong Yang^1,4^, Tianqi Liu^1,5^, Yumeng Ran^1^,*

*Bo Chen^1^, Wei Zhao^6^, Jiabo Di^1^, Zaozao Wang^1^, Jun Zhang^7*^, Xiangqian Su^1,8*^, Beihai Jiang^1*^*

*Corresponding authors.

**Supplementary Table S3:** Clinical information for 71 CRC samples, related to Figure 7B.

| Sample ID | Gender | Age | TNM stage | Pathologic stage | Differentiation grade | Preoperative treatment |  |
| --- | --- | --- | --- | --- | --- | --- | --- |
| 1 | M | 64 | T3N0M0 | IIA | Middle | NO |  |
| 2 | F | 72 | T3N0M0 | IIA | Middle | NO |  |
| 3 | F | 48 | T3N0M0 | IIA | Middle | NO |  |
| 4 | M | 67 | T3N0M0 | IIA | Middle | NO |  |
| 5 | M | 71 | T3N2bM0 | IIIC | Middle | NO |  |
| 6 | M | 73 | T3N2aM0 | IIIB | Middle | NO |  |
| 7 | M | 70 | T3N2bM0 | IIIC | Middle | NO |  |
| 8 | M | 60 | T3N0M0 | IIA | Middle | NO |  |
| 9 | M | 59 | T3N2bM0 | IIIC | Middle | NO |  |
| 10 | F | 62 | T3N1aM0 | IIIB | Middle | NO |  |
| 11 | F | 71 | T3N1aM0 | IIIB | Middle | NO |  |
| 12 | F | 62 | T3N2aM1c | IIIB | Low | NO |  |
| 13 | M | 83 | T3N0M0 | IIA | Low | NO |  |
| 14 | F | 46 | T3N0M0 | IIA | Middle | NO |  |
| 15 | F | 55 | T3N1aM0 | IIIB | Middle | NO |  |
| 16 | F | 89 | T3N0M0 | IIA | Middle | NO |  |
| 17 | F | 47 | T3N0M0 | IIA | Middle | NO |  |
| 18 | M | 61 | T4bN0M1a | IVA | Middle | NO |  |
| 19 | M | 59 | T3N1cM0 | IIIB | Middle | NO |  |
| 20 | F | 62 | T3N1aM0 | IIIB | Low | NO |  |
| 21 | F | 71 | T3N1aM0 | IIIB | Middle | NO |  |
| 22 | F | 62 | T3N1cM0 | IIIB | Middle | NO |  |
| 23 | M | 83 | T3N0M0 | IIA | Middle | NO |  |
| 24 | F | 46 | T3N0M0 | IIA | Low | NO |  |
| 25 | F | 55 | T4aN2bM0 | IIIC | Middle | NO |  |
| 26 | F | 89 | T3N0M0 | IIA | Middle | NO |  |
| 27 | F | 47 | T3N1aM0 | IIIB | Middle | NO |  |
| 28 | M | 61 | T4aN0M0 | IIB | Middle | NO |  |
| 29 | M | 64 | T3N0M0 | IIA | Middle | NO |  |
| 30 | F | 47 | T3N1bM0 | IIIB | Middle | NO |  |
| 31 | M | 38 | T3N0M0 | IIA | Middle | NO |  |
| 32 | M | 59 | T3N1bM0 | IIIB | Low | NO |  |
| 33 | F | 56 | T3N0M0 | IIA | Middle | NO |  |
| 34 | F | 59 | T3N0M1b | IVB | Low | NO |  |
| 35 | M | 58 | T3N1bM0 | IIIB | Middle | NO |  |
| 36 | M | 71 | T3N2aM0 | IIIB | Low | NO |  |
| 37 | M | 63 | T4aN1aM0 | IIIB | Middle | NO |  |
| 38 | F | 81 | T3N0M0 | IIA | Middle | NO |  |
| 39 | M | 64 | T3N2bM0 | IIIC | Low | NO |  |
| 40 | F | 47 | T3N1bM0 | IIIB | Low | NO |  |
| 41 | M | 38 | T3N2aM0 | IIIB | Middle | NO |  |
| 42 | M | 59 | T3N1bM0 | IIIB | Middle | NO |  |
| 43 | F | 56 | T4aN2aM0 | IIIC | Low | NO |  |
| 44 | F | 59 | T3N0M0 | IIA | Middle | NO |  |
| 45 | M | 58 | T3N0M0 | IIA | Middle | NO |  |
| 46 | M | 71 | T4aN0M0 | IIB | Middle | NO |  |
| 47 | M | 63 | T3N0M0 | IIA | Middle | NO |  |
| 48 | F | 81 | T3N0M0 | IIA | Middle | NO |  |
| 49 | M | 66 | T3N0M0 | IIA | Middle | NO |  |
| 50 | M | 66 | T3N0M0 | IIA | Middle | NO |  |
| 51 | M | 75 | T3N0M0 | IIA | Middle | NO |  |
| 52 | M | 44 | T3N1bM0 | IIIB | Middle | NO |  |
| 53 | F | 28 | T3N0M0 | IIA | Low | NO |  |
| 54 | F | 61 | T3N0M0 | IIA | Middle | NO |  |
| 55 | M | 55 | T3N0M0 | IIA | Middle | NO |  |
| 56 | M | 82 | T3N0M0 | IIA | Middle | NO |  |
| 57 | F | 40 | T3N0M0 | IIA | Low | NO |  |
| 58 | M | 56 | T2N0M0 | I | Middle | NO |  |
| 59 | M | 66 | T3N0M0 | IIA | Middle | NO |  |
| 60 | | M | 66 | T3N0M0 | IIA | Middle | NO |
| 61 | | M | 75 | T3N0M0 | IIA | Middle | NO |
| 62 | | M | 44 | T2N0M0 | IIA | Middle | NO |
| 63 | | F | 28 | T3N2aM0 | IIIB | Middle | NO |
| 64 | | F | 61 | T3N2aM0 | IIIB | Low | NO |
| 65 | | M | 55 | T3N2aM0 | IIIB | Middle | NO |
| 66 | | M | 82 | T3N1bM0 | IIIB | Low | NO |
| 67 | | F | 40 | T3N0M0 | IIA | Middle | NO |
| 68 | | M | 56 | T3N0M0 | IIA | Middle | NO |
| 69 | | M | 64 | T3N0M0 | IIA | Low | NO |
| 70 | | F | 72 | T3N2bM0 | IIIC | Low | NO |
| 71 | M | 56 | T3N0M0 | IIA | Middle | NO |  |
