# Supplementary material for: STK25 Loss Augments Anti‐PD‐1 Therapy Efficacy by Regulating PD‐L1 Stability in Colorectal Cancer
Source: Adv Sci (Weinh). 2025 Jul 29;12(39):e03891. doi: 10.1002/advs.202503891 (PMC12533155; doi:10.1002/advs.202503891)
Supplement: Supplementary file 7 — Supplemental Table 5 [file ADVS-12-e03891-s001.docx]

STK25 Loss Augments Anti-PD-1 Therapy Efficacy by Regulating PD-L1 Stability in Colorectal Cancer

*Xiaowen Qiao^1^*^†^*, Pu Xing^1,2^*^†^*, Hao Hao^1^, Jiangbo Chen^1^, Lin Song^1^,Yifan Hou^1^, Xinying Yang^1^, Kai Weng^1^, Jie Chen^3^, Pin Gao^1^, Tongkun Song^1^, Hong Yang^1,4^, Tianqi Liu^1,5^, Yumeng Ran^1^,*

*Bo Chen^1^, Wei Zhao^6^, Jiabo Di^1^, Zaozao Wang^1^, Jun Zhang^7*^, Xiangqian Su^1,8*^, Beihai Jiang^1*^*

*Corresponding authors.

**Supplementary Table S5.** Antibodies.

| Antibody | Manufacture | Item number |
| --- | --- | --- |
| STK25 | Proteintech | Cat#25821-1-AP |
| STK25 | Santa Cruz | Cat#sc-271196 |
| PD-L1 | Cell Signaling Technology | Cat#13684 |
| Phospho-PD-L1(Ser283) | Biorbyt | Cat#1150438 |
| Ki67 | Cell Signaling Technology | Cat#12202 |
| Flag | Sigma-Aldrich | Cat# F1804 |
| Ubiquitin | Santa Cruz | Cat#sc-8017 |
| β-actin | Sigma-Aldrich | Cat#A1978 |
| FITC anti-human CD3 | BioLegend | Cat#317305 |
| PerCP/Cyanine5.5 anti-human IFN-γ | BioLegend | Cat#502525 |
| PE anti-human/mouse Granzyme B | BioLegend | Cat#372207 |
| APC anti-human Perforin | BioLegend | Cat#308111 |
| PE/Cyanine7 anti-human CD279 | BioLegend | Cat#329917 |
| Brilliant Violet 510™ anti-human CD8 | BioLegend | Cat#344731 |
| FITC anti-mouse CD3 | BioLegend | Cat#100203 |
| PE anti-mouse CD45 | BioLegend | Cat#103106 |
| Brilliant Violet 605™ anti-mouse CD274 | BioLegend | Cat#124321 |
| PerCP/Cyanine5.5 anti-mouse CD4 | BioLegend | Cat#100433 |
| Brilliant Violet 510™ anti-mouse CD8a | BioLegend | Cat#100752 |
| PE/Cyanine7 anti-mouse CD279 (PD-1) | BioLegend | Cat#135215 |
| Violet 421™ anti-mouse Granzyme B | BioLegend | Cat#396414 |
