# Supplementary material for: STK25 Loss Augments Anti‐PD‐1 Therapy Efficacy by Regulating PD‐L1 Stability in Colorectal Cancer
Source: Adv Sci (Weinh). 2025 Jul 29;12(39):e03891. doi: 10.1002/advs.202503891 (PMC12533155; doi:10.1002/advs.202503891)
Supplement: Supplementary file 8 — Supplemental Table 6 [file ADVS-12-e03891-s009.docx]

STK25 Loss Augments Anti-PD-1 Therapy Efficacy by Regulating PD-L1 Stability in Colorectal Cancer

*Xiaowen Qiao^1^*^†^*, Pu Xing^1,2^*^†^*, Hao Hao^1^, Jiangbo Chen^1^, Lin Song^1^,Yifan Hou^1^, Xinying Yang^1^, Kai Weng^1^, Jie Chen^3^, Pin Gao^1^, Tongkun Song^1^, Hong Yang^1,4^, Tianqi Liu^1,5^, Yumeng Ran^1^,*

*Bo Chen^1^, Wei Zhao^6^, Jiabo Di^1^, Zaozao Wang^1^, Jun Zhang^7*^, Xiangqian Su^1,8*^, Beihai Jiang^1*^*

*Corresponding authors.

**Supplementary Table S6.** Relevant primer sequences.

| Gene | Primer sequences | | Species |
| --- | --- | --- | --- |
| STK25 | Forward  Reverse | 5′- GCTCCTACCTAAAGAGCACCA -3′  5′- TGGCAATGTATGTCTCCTCCAG -3′ | Human |
| PD-L1 | Forward  Reverse | 5′- GCTGCACTAATTGTCTATTGGGA - 3′  5′- AATTCGCTTGTAGTCGGCACC -3′ | Human |
| PDCD1 | Forward  Reverse | 5′- CCAGGATGGTTCTTAGACTCCC -3′  5′- TTTAGCACGAAGCTCTCCGAT-3′ | Human |
| IL-2 | Forward  Reverse | 5′- TACAAGAACCCGAAACTGACTCG -3′  5′- ACATGAAGGTAGTCTCACTGCC -3′ | Human |
| IFNG | Forward  Reverse | 5′- TCGGTAACTGACTTGAATGTCCA-3′  5′- TCGCTTCCCTGTTTTAGCTGC -3′ | Human |
| Perforin1 | Forward | 5′- GGCTGGACGTGACTCCTAAG -3′ | Human |
|  | Reverse | 5′- CTGGGTGGAGGCGTTGAAG -3′ |  |
| GAPDH | Forward | 5′- CTGGGCACTGAGCACC -3′ | Human |
|  | Reverse | 5′- AAGTGGTCGTTGAGGGCAATG-3′ |  |
| STK25 | Forward | 5′- CACCAGCATTCTCGTGTGGA -3′ | Mouse |
|  | Reverse | 5′- GTGTGGTTGTCGATCCCCT -3′ |  |
| GAPDH | Forward  Reverse | 5′- AGGTCGGTGTGAACGGATTTG -3′  5′- TGTAGACCATGTAGTTGAGGTCA -3′ | Mouse |
