# Supplementary material for: STK25 Loss Augments Anti‐PD‐1 Therapy Efficacy by Regulating PD‐L1 Stability in Colorectal Cancer
Source: Adv Sci (Weinh). 2025 Jul 29;12(39):e03891. doi: 10.1002/advs.202503891 (PMC12533155; doi:10.1002/advs.202503891)

The whole uncropped images of the original western blots

Figure 1

D

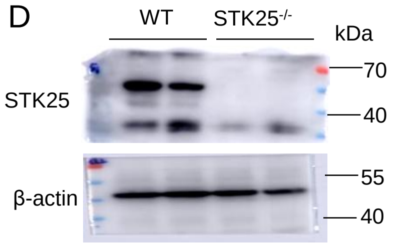

Figure 4

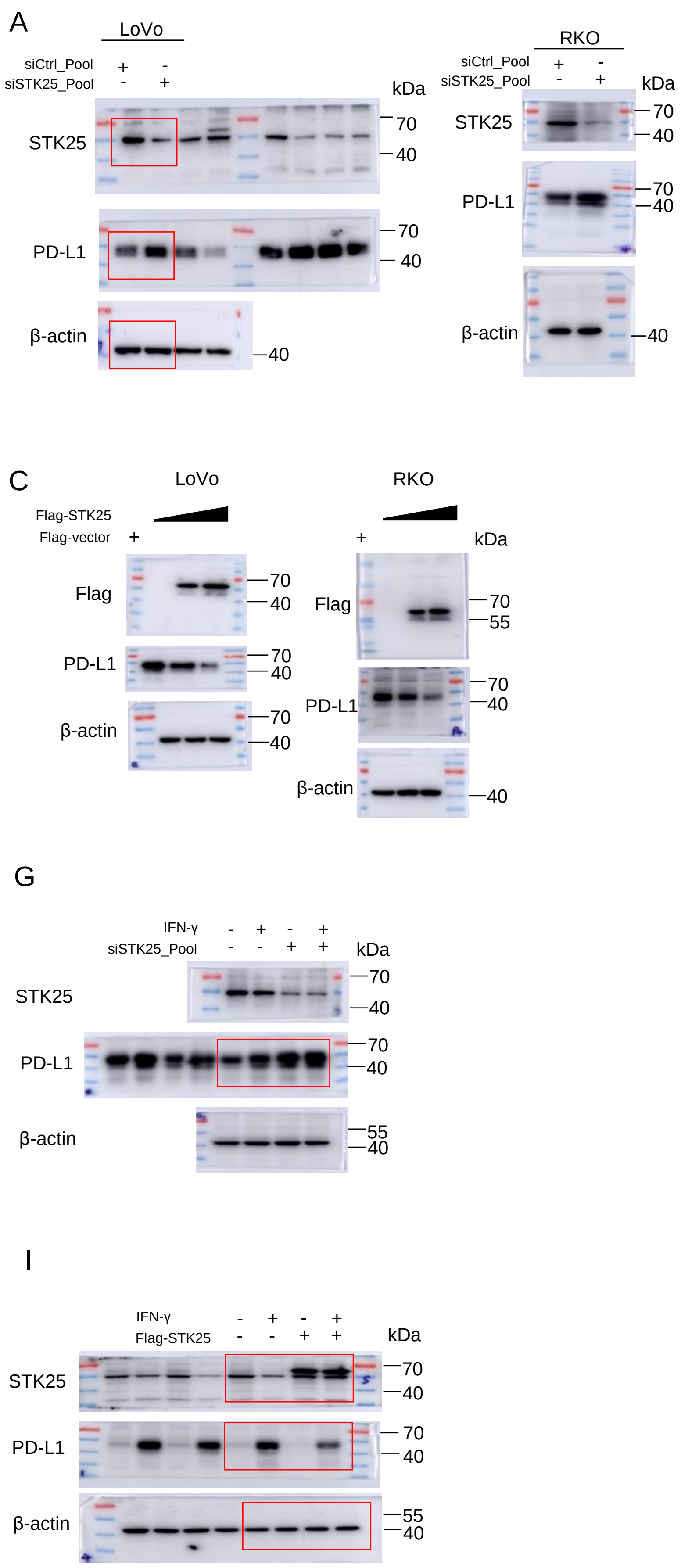

A
LoVo
siCtrl_Pool + -
siSTK25_Pool - +
STK25
PD-L1
β-actin
kDa
70
40
RKO
siCtrl_Pool + -
siSTK25_Pool - +
C
LoVo
RKO
Flag-STK25
Flag-vector +
Flag
55
G
IFN-γ - + - +
siSTK25_Pool - - + +
I
IFN-γ - + - +
Flag-STK25 - - + +

Figure 5

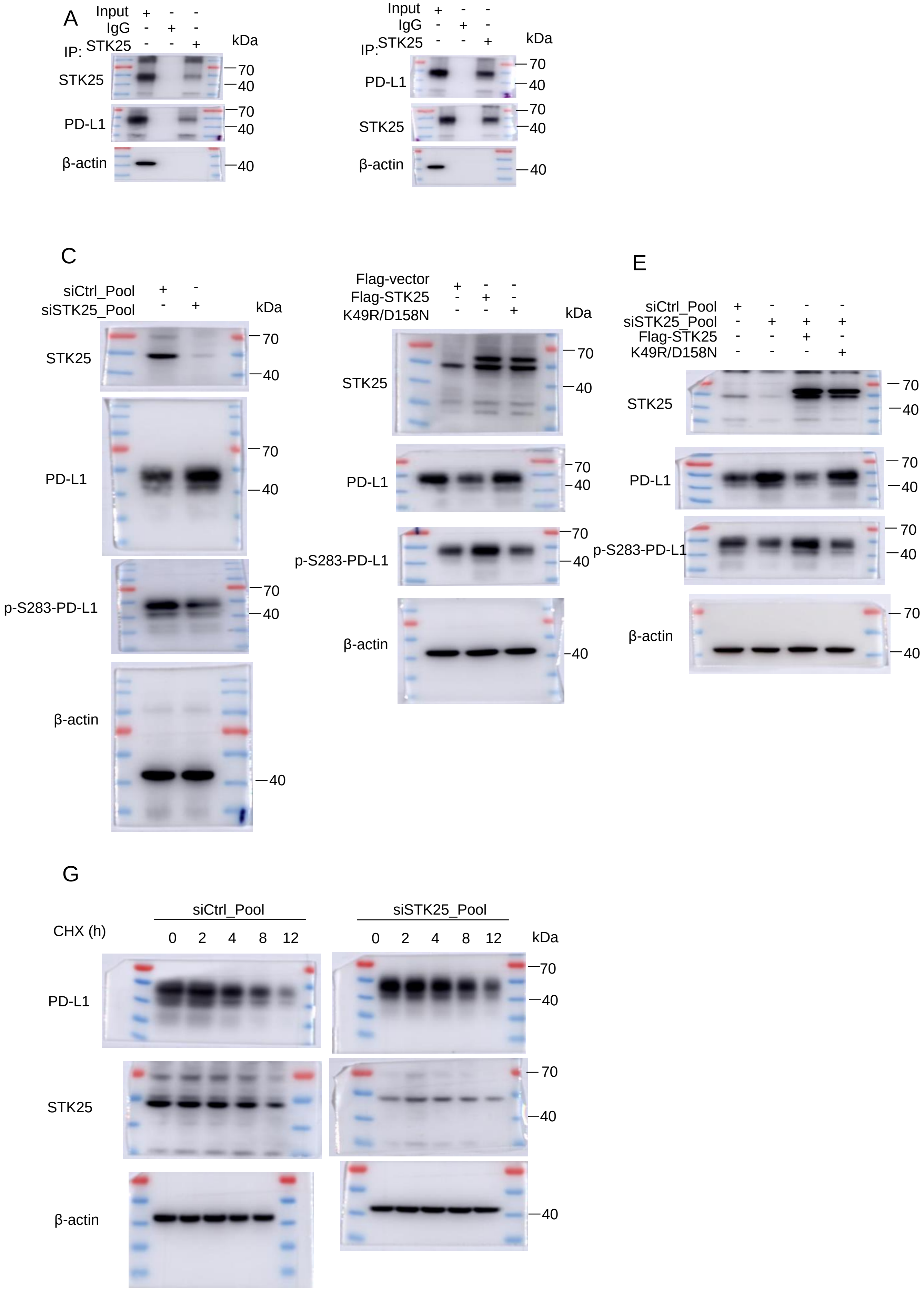
A
Input + - -
IgG - + -
IP: STK25 - - +
kDa
STK25
PD-L1
β-actin
70
40
Input + - -
IgG - + -
IP: STK25 - - +
kDa
PD-L1
STK25
β-actin
C
siCtrl_Pool + -
siSTK25_Pool - +
kDa
STK25
PD-L1
p-S283-PD-L1
β-actin
Flag-vector + - -
Flag-STK25 - + -
K49R/D158N - - +
kDa
E
siCtrl_Pool + - - -
siSTK25_Pool - + + +
Flag-STK25 - - + -
K49R/D158N - - - +
G
siCtrl_Pool
siSTK25_Pool
CHX (h)
0 2 4 8 12
0 2 4 8 12
kDa

Figure 5 (continued)

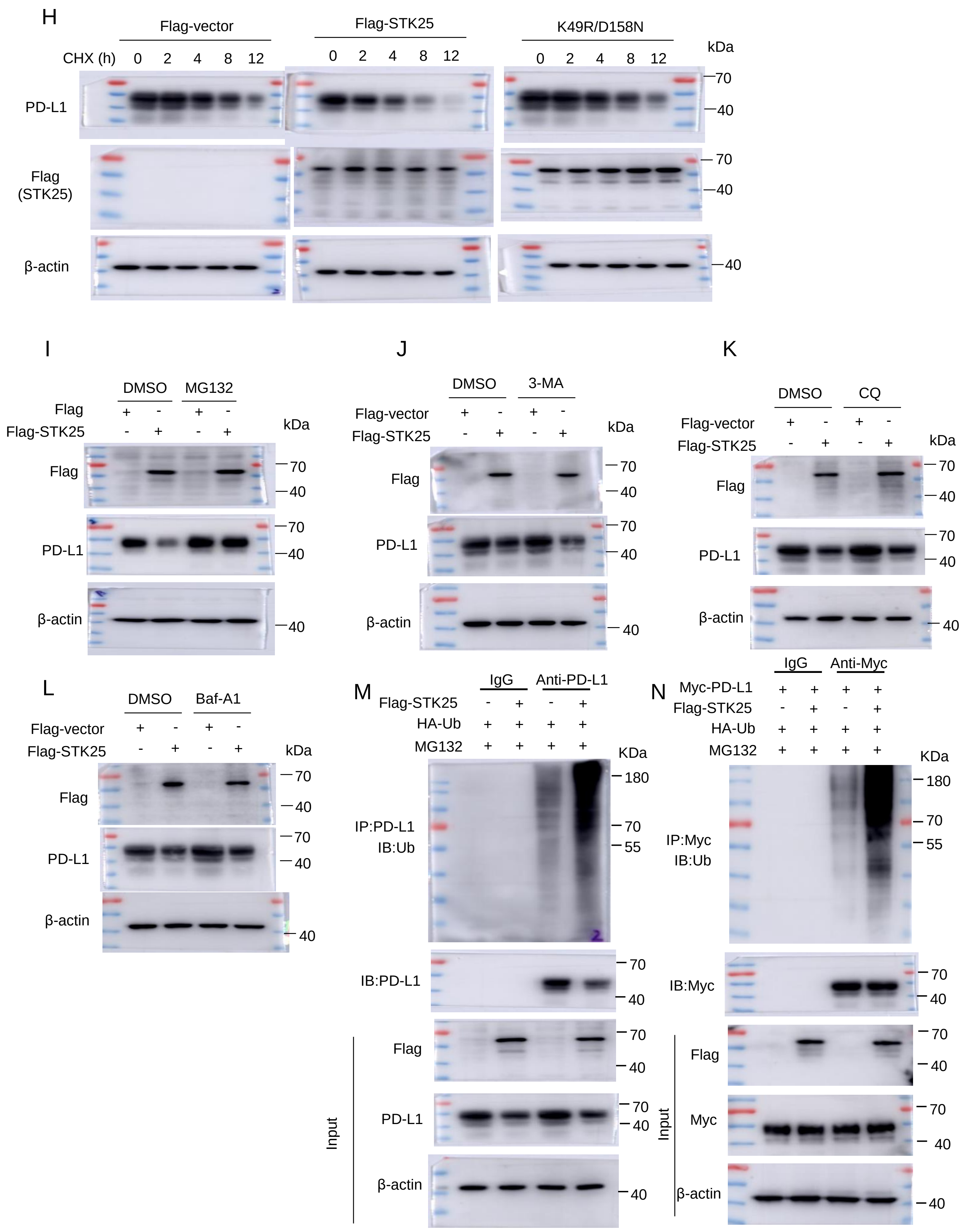

Figure 5 (continued)

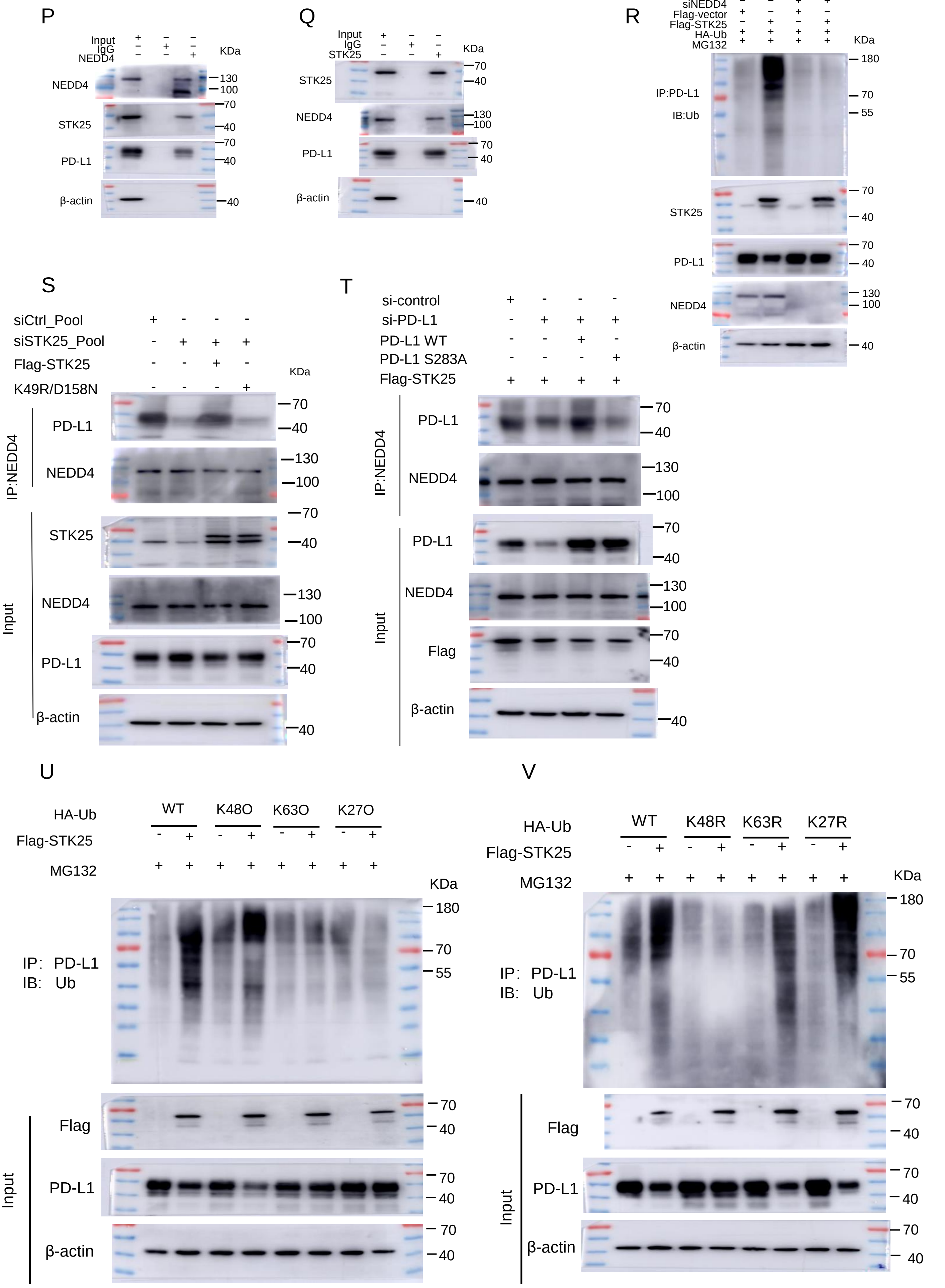

Figure 6

B

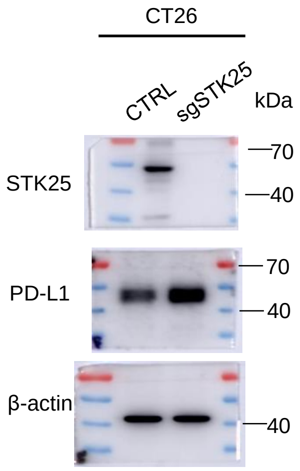

Figure 7

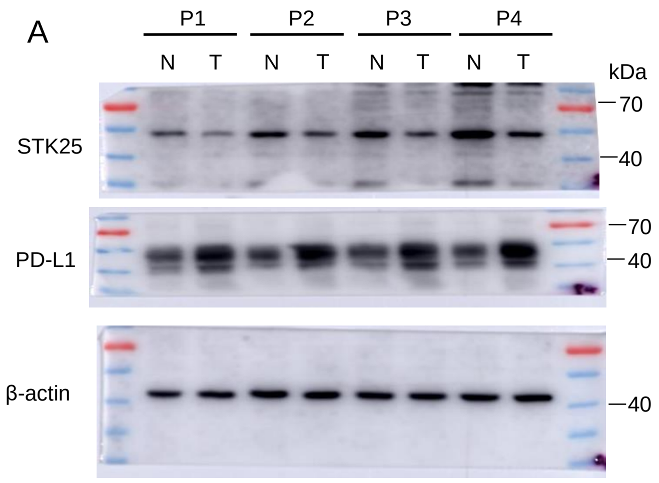

Supplemental Figure 3

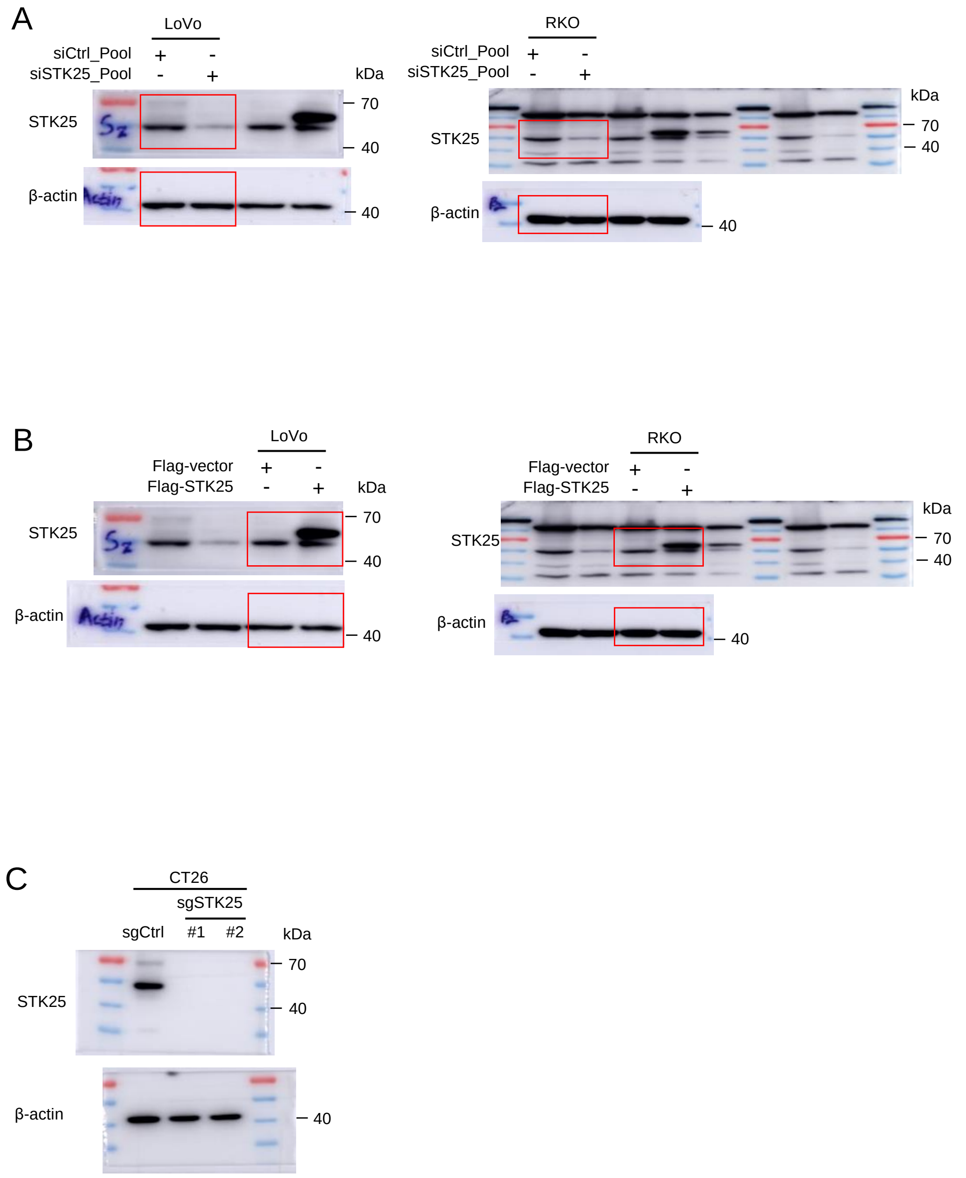

Supplemental Figure 4

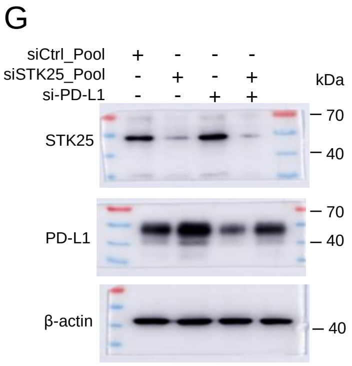

Supplemental Figure 5

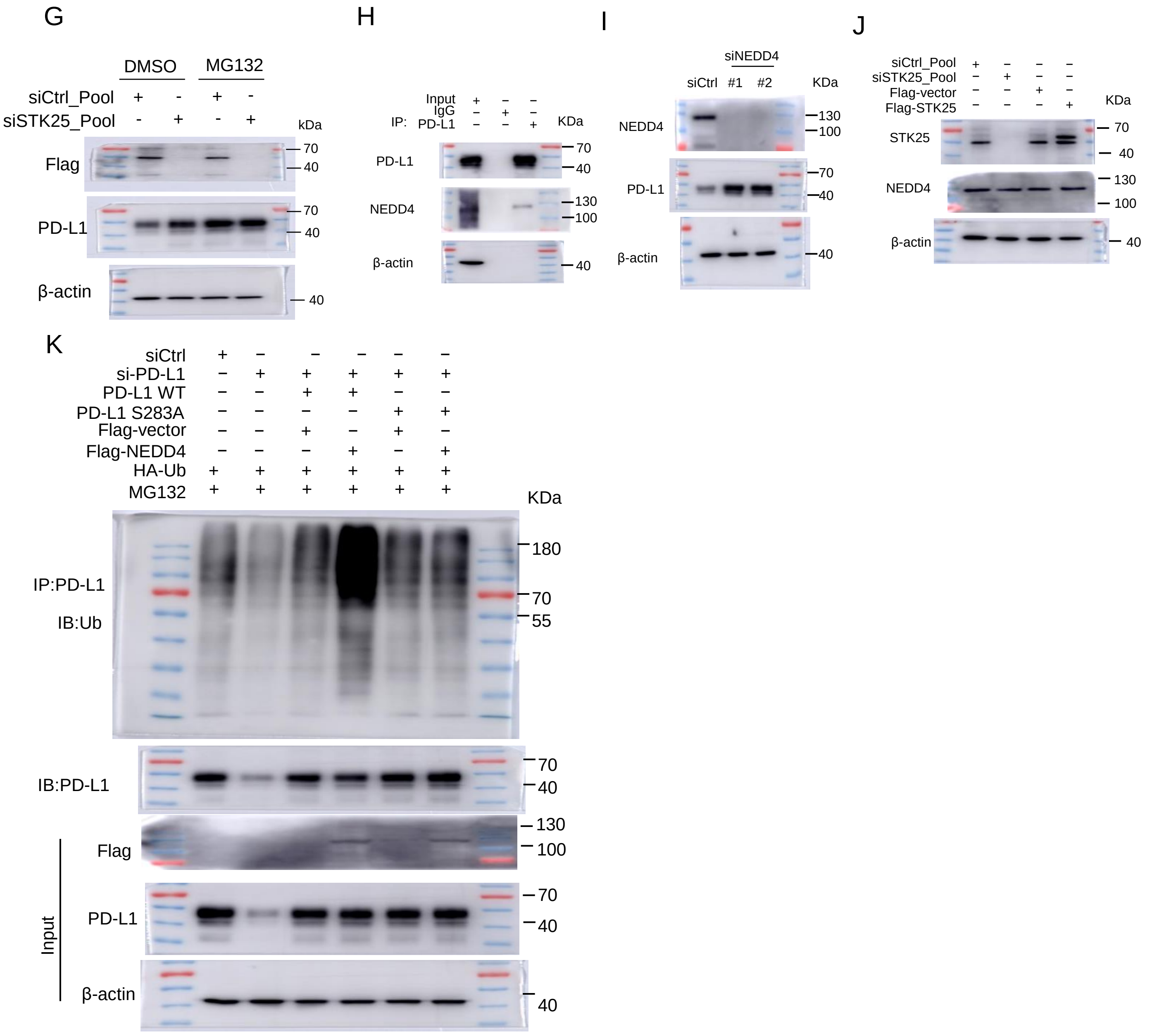

Supplement: Supplementary file 9 — Supporting Information [file ADVS-12-e03891-s006.zip › The whole uncropped images of the original western blots.pdf]
